# A high-throughput yeast approach to characterize aquaporin permeabilities: Profiling the Arabidopsis PIP aquaporin sub-family

**DOI:** 10.3389/fpls.2023.1078220

**Published:** 2023-01-19

**Authors:** Michael Groszmann, Annamaria De Rosa, Weihua Chen, Jiaen Qiu, Samantha A. McGaughey, Caitlin S. Byrt, John R. Evans

**Affiliations:** ^1^ Australian Research Council (ARC) Centre of Excellence for Translational Photosynthesis, Research School of Biology, Australian National University, Canberra, ACT, Australia; ^2^ Australian Research Council (ARC) Centre of Excellence in Plant Energy Biology, School of Agriculture, Food and Wine, University of Adelaide, Glen Osmond, SA, Australia

**Keywords:** aquaporin (AQP), PIP, membrane channel proteins, high-throughput (HT) screening, heterologous yeast expression, protein engineering

## Abstract

**Introduction:**

Engineering membrane transporters to achieve desired functionality is reliant on availability of experimental data informing structure-function relationships and intelligent design. Plant aquaporin (AQP) isoforms are capable of transporting diverse substrates such as signaling molecules, nutrients, metalloids, and gases, as well as water. AQPs can act as multifunctional channels and their transport function is reliant on many factors, with few studies having assessed transport function of specific isoforms for multiple substrates.

**Methods:**

High-throughput yeast assays were developed to screen for transport function of plant AQPs, providing a platform for fast data generation and cataloguing of substrate transport profiles. We applied our high-throughput growth-based yeast assays to screen all 13 Arabidopsis PIPs (AtPIPs) for transport of water and several neutral solutes: hydrogen peroxide (H2O2), boric acid (BA), and urea. Sodium (Na+) transport was assessed using elemental analysis techniques.

**Results:**

All AtPIPs facilitated water and H2O2 transport, although their growth phenotypes varied, and none were candidates for urea transport. For BA and Na+ transport, AtPIP2;2 and AtPIP2;7 were the top candidates, with yeast expressing these isoforms having the most pronounced toxicity response to BA exposure and accumulating the highest amounts of Na+. Linking putative AtPIP isoform substrate transport profiles with phylogenetics and gene expression data, enabled us to align possible substrate preferences with known and hypothesized biological roles of AtPIPs.

**Discussion:**

This testing framework enables efficient cataloguing of putative transport functionality of diverse AQPs at a scale that can help accelerate our understanding of AQP biology through big data approaches (e.g. association studies). The principles of the individual assays could be further adapted to test additional substrates. Data generated from this framework could inform future testing of AQP physiological roles, and address knowledge gaps in structure-function relationships to improve engineering efforts.

## Introduction

Deciphering the function of membrane transporters is essential for protein engineering and design, aiding development of designer proteins for a multitude of industries, such as medical, chemical, environmental and food (Xu et al., 2020; Horne and Shukla, 2022). Improved understanding of membrane transporter function is also important for ensuring sustainable food production, with membrane transporters being key targets for improving plant water and nutrient uptake efficiency (Schroeder et al., 2013). Although advances have been made in intelligent-design approaches, significant challenges remain in mapping amino acid sequences to protein properties and biological activities due to limitation in available experimental data to inform association studies and modeling approaches (Horne and Shukla, 2022). Generation of large-scale cataloguing of transport functions across diverse sets of a given transporter type is fundamental for addressing these challenges.

Aquaporins (AQPs) constitute a major family of channel proteins with great potential to address a multitude of industry challenges (Tang et al., 2015; Hélix-Nielsen, 2018; Jafarinejad, 2020) and interesting targets for engineering more resilient and productive crops (Afzal et al., 2016; Singh et al., 2020). AQPs are found across all phylogenetic kingdoms and help facilitate the diffusion of substrates across biological membranes (Chaumont and Tyerman, 2017). In plants, AQPs are implicated in numerous key physiological processes including: water relations, organ growth, fertilisation, seed development and germination, abiotic stress responses, defence signalling, nutrient uptake and tolerance, and photosynthesis (Chaumont and Tyerman, 2017). Such diversity in functional roles is enabled by their wide-ranging permeability to many substrates indispensable for plant growth such as: water, CO_2_ and nitrogen (NH_3_/ ${\text{NH}}_{4}^{+}$ ,urea and nitrate); micronutrients (boric acid and silicic acid) and other metalloids; signalling molecules hydrogen peroxide (H_2_O_2_) and nitric oxide (NO); O_2_ and lactic acid to cope with anoxic stress; and key nutrients such as potassium (Chaumont and Tyerman, 2017; Singh et al., 2020; Qiu et al., 2020).

The AQP gene family has expanded the most in plants, providing a rich source of sequence diversity to inform structure-function relationships. This diversity likely reflects greater duplication rates of plant genomes and the adaptive potential AQPs provide for a sessile lifestyle. Originating from only a few distinct isoforms in green algae, the genomes of Angiosperm species commonly harbour between 30-50 isoforms, with extremes of 84 and 121 in tobacco and canola, respectively (Groszmann et al., 2017; Sonah et al., 2017; De Rosa et al., 2020; Groszmann et al., 2021). There are up to 13 AQP subfamilies recognised in the plant kingdom, the PIP, TIP, NIP, SIP, and XIP subfamilies predominate the angiosperms, GIP and HIP subfamilies only occur in older plant lineages (e.g. mosses), LIPs are exclusive to diatoms, and the ancestral MIPs A-E are unique to green algae (Laloux et al., 2018). Each subfamily is generally characterised by sequence composition, a tendency to localise to different subcellular membranes, and transport different sets of substrates.

AQP monomers form a characteristic hour-glass membrane-spanning pore differing in aperture and residue composition which, in large part, determines their particular substrate selectivity and permeabilities. Four AQP monomers assemble to form tetrameric complexes which create a fifth central pore implicated in the movement of CO_2_ (Kaldenhoff et al., 2014) and ions (Yu et al., 2006) across membranes. Key pore features such as the dual Asn-Pro-Ala (NPA) motifs, the aromatic/Arginine (ar/R) filter and Froger’s position have been associated with broader substrate selectivity (e.g. water vs. urea). However, gaining a more nuanced understanding of signatures related to substrate selectivity, transport efficiency, and substrate exclusivity between isoforms, requires more detailed and larger scale characterisations. While a single AQP isoform can permeate a variety of substrates, surprisingly few have been surveyed for multiple substrates in parallel under similar conditions to establish catalogues of comparative transport profiles.

Sufficiently accurate and high-throughput phenotyping of AQP transport remains a major bottleneck for building of a functionality catalogue. Transport function of AQPs is routinely assessed in heterologous systems such as oocytes, liposomes, artificial membranes, and yeast (Madeira et al., 2016). Most of these systems and assays require specialized equipment (e.g. stopped-flow spectrophotometer), or complicated setups (e.g. artificial polymer membranes), or are labor intensive (e.g. *Xenopus laevis* oocytes), which preclude their use for high-throughput applications. By contrast, yeast offer a simple and versatile host for the heterologous production of aquaporins (Öberg et al., 2009; Bill, 2014), with which to test different substrates.

The budding yeast, *Saccharomyces cerevisiae*, is a robust model used in chemical sensitivity assays for drug discovery and identifying actions of small molecules *in vivo* (Denny and Steel, 2015; Denny, 2018). Most commonly, such screens monitor growth inhibition/promotion of the treated yeast as a reliable easy to measure proxy for chemical uptake and action. This premise has been used in a growing list of AQP studies whereby the altered growth response of the AQP expressing yeast correlates with enhanced intracellular accumulation of the tested substrate (Bienert et al., 2007; Bienert et al., 2008; Dynowski et al., 2008b; Fitzpatrick and Reid, 2009; Bienert et al., 2011; Kumar et al., 2014; Mao and Sun, 2015; To et al., 2015; Mosa et al., 2016; Rhee et al., 2017; Wang et al., 2019). The diversity of well characterized mutant strains of *S. cerevisiae* with enhanced sensitivity and growth responses to substrate uptake and accumulation, enables bespoke optimization for screening specific substrate permeabilities of heterologously expressed AQPs. Mutant strains are available that are hyper-sensitive to a given cytotoxic agent, or where native transporters for compounds essential for growth are not functional and require alternative uptake routes, such as a heterologously expressed AQP.

Altered growth phenotypes of AQP-expressing yeast in response to treatment can be detected through cell dilution spot tests for colony formation on solid medium containing the test substrate. While this traditional approach gives a visual indication of relative differences in phenotypes, the assessment of differences is subjective (Hung et al., 2018). Real-time optical density (OD) monitoring of yeast micro-volume cultures (< 300 μl) can overcome the limitations of agar-based spot assays. They are particularly suitable for detecting small phenotypic changes in yeast population growth, the detection of relative differences is impartial and the approach for monitoring responses to chemical treatments is well-established (Warringer and Blomberg, 2003; Toussaint et al., 2006; Marešová and Sychrová, 2007).

Here, we establish a methodological framework that addresses the phenotyping bottleneck of determining putative AQP substrates. This high-throughput micro-cultivation yeast system enables precise characterization of growth phenotypic responses of AQP-expressing yeast upon exposure to treatments in order to infer solute transport. We applied this framework to all 13 members of the Arabidopsis PIP aquaporin family (AtPIPs), determining candidates for water, hydrogen peroxide, boric acid and urea transport. Sodium permeability for all AtPIPs was investigated though elemental analysis techniques.

This approach can be used to efficiently catalogue the transport functions of many AQPs to help clarify their biological roles in plants and for use in associations studies to inform structure-function relationships towards improved protein engineering efforts.

## Materials and methods

Detailed material and methods are provided as **Supplemental Information**. Briefly, *AtPIP* and control gene coding sequences were commercially synthesised (Genscript) as gateway-enabled entry constructs and cloned into destination vectors from the Advanced Gateway^®^ series of yeast expression plasmids (Alberti et al., 2007) to create the various yeast expression clones. These were transformed into appropriate yeast strains using Frozen-EZ yeast Transformation Kit II (Zymo Research). *AtPIP-GFP* were used to evaluate heterologous AtPIP production, with GFP signal detected in concentrated yeast cultures using the Infinite M1000 Pro plate reader (TECAN). Subcellular localization in yeast cells was performed using confocal microscopy on a Zeiss LSM780 confocal laser-scanning microscope (Carl Zeiss) operated by Zen Black software. Quantification of AtPIP2;5 interactions with AtPIP1 proteins using the Y2H mbSUS was performed as per (Grefen et al., 2007). Yeast spheroplasts were generated using zymolyase digestion (Zymo Research) and spheroplast bursting due to osmotic shock measured using a Cary 60 UV-VIS (Agilent) spectrophotometer with OD_650_ reading at 0.1 sec intervals. Micro-volume yeast cultures were cultivated and OD readings measured using a Spectrostar Nano microplate reader (BMG, Germany) in Nunc-96 400 µL flat bottom untreated 96-well plates (Thermo Scientific Cat#243656) with lid and 200μl culture volume per well. Default cycling conditions for yeast growth assays were: 250 cycles at 10 mins per cycle (total time ~42-50 hrs); incubated at 30°C with a slightly warmer lid; shaking frequency of 400 rpm in double orbital shaking mode; 5 mins shaking per cycle prior to the OD reading, with the remaining time the plate sitting idle on the incubation plate; OD readings invoke orbital averaging at scan diameter of 4mm and 22 flashes per well, recording at 650nm. OD_650_ readings minus the blank were corrected for non-linearity using our pre-determined calibration function to generate a ‘true’OD_650_ at a 1cm path-length. The data was then converted into growth curves that were smoothed using several filters illustrated in **Figure S14** to obtain ^Corr.^OD_650_ values. These were finally log (LN) transformed using ^Corr.^OD_650_ at time ‘t’ divided by the initial starting OD (^Corr.^OD_i_), details of these corrections are described in **Supplemental Materials and Methods** section ‘Processing of growth curves: generating Ln(^Corr.^OD_t/_^Corr.^OD_i_) values’. Specifics of freeze-thaw, H_2_O_2_, boric acid, urea, and NaCl treatments are detailed in **Supplemental Materials and Methods**.

## Results

### Developing high-throughput micro-volume yeast culturing assays to assess aquaporin function

#### Optimizing conditions for reproducible growth curves

**Figure 1:**
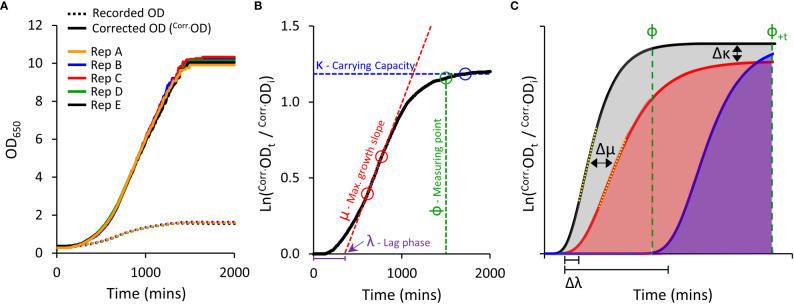
Yeast micro-cultivation setup and growth data outputs. **(A)**, Optimised micro-cultivation conditions produce repeatable growth curves of replicate cultures spaced across a 96-well plate. The growth curves of recorded OD values are compressed due to the progressive non-linear response of optical detection. Applying a calibration function produces corrected OD values (^Corr.^OD) and a more accurate representative growth curve. **(B)**, A yeast population growth curve (Ln ^Corr.^Od_t_/^Corr.^Od_i_) depicting the three major derived growth traits (λ, μ, and κ) and the dynamic standardizing measuring point, Phi (ф). **(C)**, Conceptual examples demonstrating the use of Area Under the Curve (AUC) as a measure of cumulative growth differences. Untreated yeast population growth (black) and two treatment growth scenarios (blue and red). Ф is allocated to the untreated growth curve. The red curve shows a slightly longer lag phase (Δλ), reduced maximum rate of growth (Δμ; differences between yellow dotted tangent lines), and lower carrying capacity (Δκ), captured as a substantially reduced AUC (shading) than that of the untreated black curve. The blue curve shows a longer lag phase, but growth rate and carrying capacity similar to untreated. No AUC is detected at ф, but AUC can be detected by shifting to ф_+t_ (note: ΔAUC will be less (underestimated) when using ф_+t_ as control population has ceased growing).

We established a high-throughput yeast micro-cultivation (200 µl) method using 96-well plates. The micro-cultures were incubated in a plate reader with versatile control over temperature, shaking, and OD reading modes. We optimized these parameters to find conditions that generated repeatable growth curves (**Figure 1A**; see **Supplemental Materials and Methods** for details). We observed that micro-volume cultures tended to aggregate and sediment in wells regardless of the shaking intensity. Sedimentation was managed using a double orbital shaking mode which dispersed yeast evenly across the bottom of the well and recording OD as an average of multiple measurements at distinct points around each well using the well scanning mode on the plate reader.

#### Adjusting for non-linearity of OD measurements at high cell density

Growing yeast cultures quickly achieve densities that far exceed saturation limits of optical detection in spectrophotometers (**Figure 1A**) (Stevenson et al., 2016). This severely underestimates ‘true’ ODs at higher cell densities, resulting in compressed growth curves and systematic distortion of extracted fitness components required to evaluate culture health and growth (Warringer and Blomberg, 2003; Fernandez-Ricaud et al., 2016).

We compared ‘recorded’ ODs against ‘true’ ODs calculated from dilution factors. A single polynomial function (y=1.9481x^4^-4.2474x^3^+5.0329x^2^+0.3441x) described the relationship between ‘recorded’ and ‘true’ OD datasets that was valid for all *S. cerevisiae* strains used in this study (R^2^ > 0.99; **Supplemental Figure S1**); noting this relationship will be spectrophotometer-dependent. Further transformation of the ‘true’ OD was undertaken (illustrated in **Figure S14**), to obtain correct OD values (^Corr.^OD), generating uncompressed growth curves with improved resolution of key derived growth characteristics: initial lag phase (λ), maximum growth rate (μ), and final carrying capacity or biomass yield (κ) (**Figures 1A, B**).

Examination of growth curves with and without OD correction, revealed that the calibration function does not introduce artifacts that could mislead interpretation of growth curve results. Rather, growth-curve calibration provides a more realistic representation of the health of the culture. When using raw ‘recorded’ OD reads, λ was slightly under-estimated and both μ and κ were greatly under-estimated (**Supplemental Note 1**, **Supplemental Figures S12** and **S13**).

#### Establishing the Phi (ф) measuring point and AUC value

To compare growth phenotypes of various PIP-expressing yeast, we calculated the Area Under the Curve (AUC) as a single all-encompassing parameter capturing changes in λ, μ and κ (**Figure 1C**). We observed that heterologous expression of AtPIPs can differentially alter yeast growth traits independent of chemical treatment (**Supplemental Table S1**). This may occur to an even greater extent when assessing more diverse AQP isoforms from other sub-families. Altered inherent growth means yeast cultures mature at different rates, thereby complicating the evaluation of growth differences, especially when measuring all cultures at a single time point. Measuring the AUC of a given culture sub-set too soon potentially misses growth phenotypes arising from subtle responses to treatments. Measuring too late, and the rapidly growing control cultures have plateaued, allowing the slower growing treated cultures time to catch up and reduce the difference. To account for variation in culture maturity times, we implemented a dynamic standardizing measuring point termed Phi (ф), defined just prior to the stationary phase of log transformed growth curves, at the point the population growth rate drops below 5% of maximum (**Figure 1B**). ф is established on the culture growing in optimal conditions for a given AQP set (**Figure 1C**), i.e. the untreated control when evaluating cytotoxic compounds (e.g. H_2_O_2_), or the culture with the highest supplementation of essential nutrient when examining growth requiring agents (e.g. urea). AUCs for all cultures were calculated from the start of cultivation until ф (**Figure 1C**), with AUC_treated_/AUC_control_ providing relative differences in growth (ΔAUC). In our routine conditions, all control cultures reached and remained in stationary phase for an extended period of time. As such, ф can be shifted (ф_+t_) in order to capture additional data from treated cultures that grow very slowly; with an understanding that ΔAUC will be underestimated because the control culture plateaued earlier (**Figure 1C**). Once ΔAUC values are established for each AQP, relative growth phenotypes are compared between AQPs.

### Heterologous AtPIP production in yeast

Having an abundance of AQP protein in the yeast cell is the first essential requirement for robust functional evaluations and improves the detection limit in response to treatments. For example, the water permeability for AtPIP2;3 was assessed using two differentially active promoters, with greater freeze-thaw tolerance (a proxy for water permeability) achieved using the strong GPD promoter relative to the less active TPI1 promoter (**Supplemental Figure S2**). To maximize the likelihood of high AtPIP production we (i) used high copy number plasmids with minimal load burdens on yeast growth, (ii) used a strong constitutive GPD promoter with complementing terminator, (iii) ensured codon usage compatibility between AtPIPs and yeast, and (iv) modified the Kozak sequence to enhance translational initiation (see **Supplemental Materials and Methods**). A parallel collection of *AtPIP-GFP* transgenes that differed only in the C-terminal GFP fusion compared to the expression vectors used in the functional assays, were used for evaluating heterologous AtPIP production *in vivo* and subcellular localization. Of the 13 AtPIPs assessed, 12 AtPIP-GFP yeast lines repeatedly emitted strong GFP signal (440 GFP fluorescence units/cell OD1 average), indicating high AtPIP protein production (**Supplemental Figure S3**). Relative to the other PIPs, AtPIP1;4 had the lowest abundance, emitting 113 GFP fluorescence units/cell OD1, representing 27% of the average fluorescence intensity for all AtPIPs combined (415 GFP fluorescence units/cell OD1, black dotted line, **Supplemental Figure S3**). The reduced AtPIP1;4 protein abundance relative to the other AtPIPs remains unexplained, however we could still detect growth phenotypes comparable to the other AtPIPs through our functional experiments (described below).

### Subcellular localization of AtPIPs in yeast

**Figure 2:**
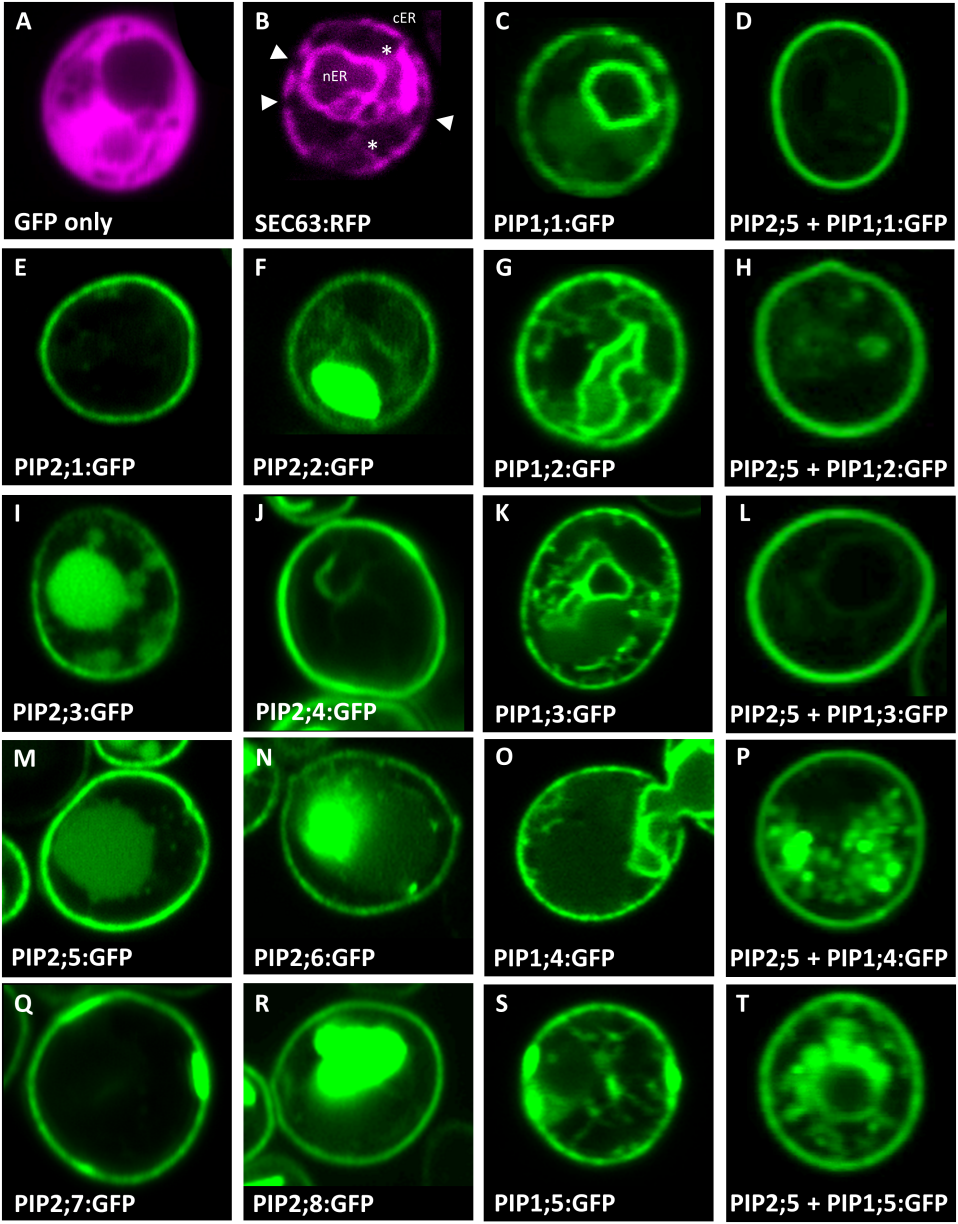
Sub-cellular localisation of AtPIPs in yeast. Confocal microscopy images of: **(A)**, an eGFP only control showing diffuse cytosolic localised signal. **(B)**, SEC63::RFP endoplasmic reticulum (ER) marker showing the prominent nuclear envelope ER domain (nER) and a peripheral or cortical ER domain (cER). The cER lies just beneath the plasma membrane but is not continuous around the perimeter with gaps distinguishing it from plasma membrane localisation (solid triangles). Cytoplasmic tubules link the two ER domains (*). **(E**, **F**, **I**, **J**, **M**, **N**, **Q** and **R)**, AtPIP2-eGFP proteins expressed alone predominantly localise in a distinct continuous ring of signal around the cell perimeter coinciding with the plasma membrane. In several cases, eGFP signal can also be detected in internal storage vacuoles. **(C**, **G**, **K**, **O** and **S)**, AtPIP1-eGFP proteins expressed alone localise to the nER, ER tubules and a patchy cER signal overlaying PM localisation. **(D**, **H**, **L**, **P** and **T)**, AtPIP1-eGFP proteins co-expressed with AtPIP2;5 with the majority of the fluorescence signal localised to the PM, similar to AtPIP2 proteins. Fluorescence signal false colored red for marker lines in A and B, and green for AtPIP-GFP lines in **(C-T)**.

In addition to ample heterologous protein production, sufficient AtPIP needs to localize to the yeast plasma membrane (PM) in order to evaluate AQP-facilitated substrate transport into the cell. Sub-cellular localization of the AtPIPs was evaluated using confocal microscopy of AtPIP-GFP lines and compared against cytosolic (GFP only) and endoplasmic reticulum (ER; SEC63-RFP) markers (**Figure 2**). Free GFP is cytosolically localized (**Figure 2A**). The SEC63-RFP marker reveals the web-like ER network, with the prominent nuclear envelope ER domain (nER) and peripheral or cortical ER domain (cER) (**Figure 2B**). The cER lies immediately adjacent to the PM but is discontinuous around the perimeter with discernible gaps, distinguishing it from PM localisation (**Figure 2B**). A sharp ring around the cell perimeter was seen for all 8 AtPIP2-GFP proteins, indicating a consistent strong targeting to the PM (**Figures 2, E, F, I, J, M, N, Q** and **R**). When expressed alone, the five AtPIP1-GFP proteins show a faint continuous ring around the periphery of the cell, consistent with PM localisation (**Figures 2, C, G, K, O** and **S**), but less efficient than observed in the AtPIP2s. In addition to localizing to the cell periphery, all 5 AtPIP1s show dual localization consisting of a patchy peripheral ring and internal webs like those observed in the SEC63-RFP ER marker (**Figures 2, C, G, K, O** and **S**).

### Co-expression with AtPIP2;5 enables AtPIP1s to more efficiently localize to the yeast PM

**Figure 3:**
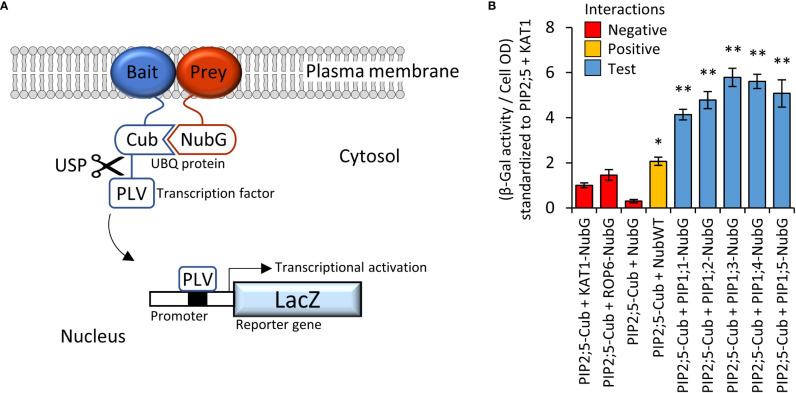
**(A)**, Illustration of mbSUS yeast two-hybrid system. The mutant N-terminal ubiquitin domain (NubG) and C-terminal ubiquitin domain (Cub) can reconstitute the full-length ubiquitin protein (UBQ) only when brought into close proximity *via* a membrane bound and interacting Bait and Prey protein combination. The reconstituted UBQ is recognised by Ubiquitin-Specific Proteases (USP), releasing the artificial transcription factor PLV (proteinA-LexA-VP16) that is translationally fused to the Cub domain. The freed PLV then enters the nucleus and activates the *LacZ* reporter gene that encodes for a β-galactosidase. **(B)**, AtPIP2;5 is capable of strong protein-protein interactions with each of the AtPIP1 isoforms. The intensity of the AtPIP2;5 (bait) and AtPIP1 (prey) interaction was assayed by measuring β-galactosidase activity *via* colorimetric monitoring of o-nitrophenyl-β-D-galactoside (ONPG) conversion to the yellow o-nitrophenol. Control lines: NubG (pNX35-DEST), a mutant Nub variant with low affinity for Cub. When linked to plasma membrane localizing Arabidopsis ROP6 or KAT1 proteins, it acts as a prey control reporting incidental UBQ reconstitution through simple random close insertion of abundantly produced membrane bound proteins. NubG expressed alone should not interact with Cub and negligible reporter activity was observed. NubWT (pNubWTXgate) is a soluble cytoplasmic localized N-terminal ubiquitin domain with a high affinity for Cub and acts as a positive control able to interact with the Cub domain of AtPIP2;5-Cub independent of bait interaction. The detected activity (orange) demonstrates that the Cub domain fused to AtPIP2;5 was accessible to Nub and USPs. Each of the AtPIP2;5 + AtPIP1 interactions (blue) significantly exceeded spurious background levels (red). All error bars are SEM. ANOVA post-hoc Fisher’s LSD versus AtPIP2;5 + KAT1, * p < 0.05, ** p < 0.01. N = 4 biological reps over 2 experimental runs.

PIP2 proteins can interact and guide PIP1 proteins more efficiently to the PM (Jozefkowicz et al., 2017). The Yeast-two-Hybrid mating-based Split-Ubiquitin System (Y2H mbSUS; **Figure 3A**) was used to screen an AtPIP interactome library. Yeast co-expressing the bait AtPIP2;5-CubPLV and any of the AtPIP1;1-Nub to AtPIP1;5-Nub prey proteins, activated the *lacZ* reporter ≥ 4-fold above background levels (**Figure 3B**), demonstrating that AtPIP2;5 strongly interacted with each AtPIP1. Co-expression of AtPIP2;5 with *GFP* tagged versions of AtPIP1;1 to 1;5, resulted in the fluorescence signal now being predominantly associated with the PM and comparable to AtPIP2 isoforms (**Figures 2, D, H, L, P** and **T**).

AtPIP2;5 was chosen because, among the AtPIP2s, its expression in yeast resulted in moderate relative levels of sensitivity to the tested substrates (compared to other PIP2s), enabling further enhancements in sensitivity due to the co-expressed AtPIP1.

### Characterizing AtPIP water permeability

Yeast are sensitive to very rapid freezing events, with the formation of intracellular ice crystals causing cell damage and death. Freeze-thaw survivorship depends on how rapidly water can efflux from the cell across the PM (Cabrera et al., 2020), which in turn correlates with the water transport function of water permeable AQPs (Tanghe et al., 2002; Tanghe et al., 2004; Soveral et al., 2006). Therefore, water permeability of AQP variants can be rapidly determined in yeast by screening for improved freeze-thaw survivorship over extended time periods (To et al., 2015). We adapted a freeze-thaw assay to our micro-cultivation setup to test the permeability of AtPIPs to water. For wild type yeast carrying an empty vector, successive freeze-thaw treatments incrementally decreased ΔAUC (**Supplemental Figures S4A and B**). Freeze-thawing prolonged the lag phase (λ) (**Supplemental Figure S4C**), consistent with a reduction in viable cell count of the starting population, which delayed detection of population growth. The sensitivity of the freeze-thaw assay was improved by using the *aquaporin* null mutant background (*aqy1 aqy2*), which is compromised in tolerance to rapid freeze-thaw events (Tanghe et al., 2002; Tanghe et al., 2004). Two freeze-thaw cycles were sufficient to essentially render the entire *aqy1 aqy2* starting population unviable (**Supplemental Figures S4A-C**). Heterologous expression of a water permeable AQP (*AtPIP2;1*) (Verdoucq et al., 2008), dramatically improved the tolerance of the *aqy1 aqy2* mutant to repeated freeze-thaw treatments (**Supplemental Figures S4A-C**).

**Figure 4:**
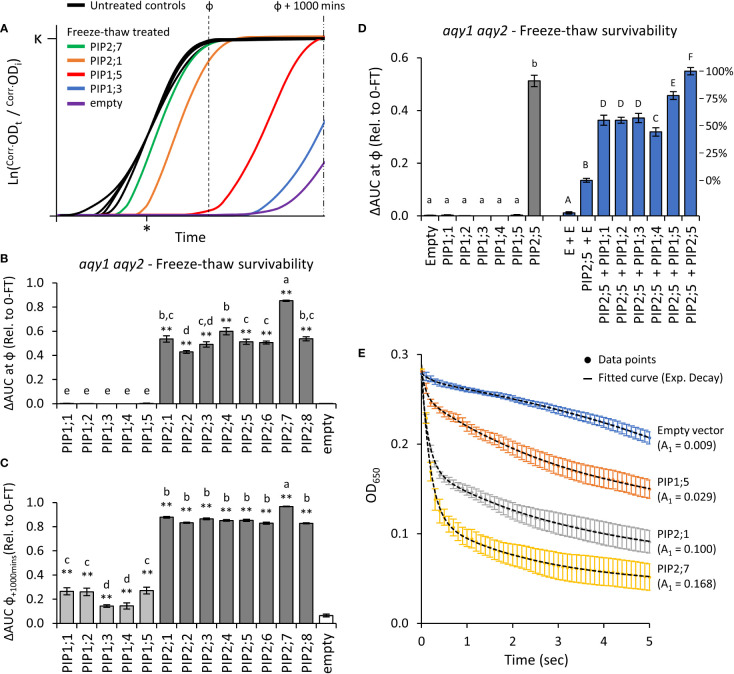
Water permeability assays using two freeze-thaw cycles with yeast expressing different AQP genes. **(A)**, Growth curves for untreated controls and following two freeze-thaw cycles. For illustrative purposes the untreated curves have been standardized to a fixed lag-point (*) and the treated curves remain relative to their respective control. **(B)**, Relative AUC for the 13 AtPIP isoforms, calculated with ф. **(C)**, Relative AUC after extended growth with AUC calculated at ф+1000. **(D)**, Relative AUC for AtPIP1s expressed singly or co-expressed with AtPIP2;5. **(E)**, Change in OD of yeast spheroplast suspensions following osmotic shock. The contribution of the rapid initial phase (A_1_ value in parentheses) reflects the permeability derived from fitted two-phase exponential curves; empty vector: y = [0.00881 • *e* ^(-x/0.243)^] + [-0.05398 • *e* ^(-x/-6.47128)^]; AtPIP1;5: y = [0.02937 • *e* ^(-x/0.09966)^] + [0.13874 • *e* ^(-x/3.76055)^]; AtPIP2;1: y = [0.10037 • *e* ^(-x/0.15797)^] + [0.10763 • *e* ^(-x/3.51469)^]; AtPIP2;7: y = [0.16814 • *e* ^(-x/0.18973)^] + [0.07791 • *e* ^(-x/2.43538)^]. All error bars are SEM. For **(B, C)**, asterisks indicate statistical difference from empty vector control, ANOVA with Fishers LSD test (* *P* < 0.05; ** *P* < 0.01); letters denote different statistical rankings, ANOVA with Tukey’s test (*P* < 0.05). For D, letters denote different statistical groupings, lowercase among single expressed and uppercase among co-expressed AtPIP yeast lines, ANOVA with Tukey’s test (*P* < 0.05). N = 12 (AtPIP1s) and 8 (AtPIP2s) across 4 experimental runs for **(B)** and **(C)**. N = 6 across 3 experimental runs for D. N = 6 across 2 experimental runs for **(E)**.

Application of two freeze-thaw treatments to *aqy1 aqy2* yeast expressing one of the 13 *AtPIP* genes or an empty vector, differentially affected the growth curves (**Figure 4A**). All of the AtPIP2 proteins had sufficient capacity to transport water across the PM to confer freeze-thaw tolerance, but their response varied with AtPIP2;7, the most, and AtPIP2;2, the least tolerant to freeze-thaw (**Figure 4B**). At ф, growth was not detected for any *AtPIP1* expressing lines. However, inspection of the raw growth curves indicated that over the course of the 42h growth period there was a low level of growth recovery post freezing treatments. By calculating AUC at ф + 1000 mins, freeze-thaw tolerance associated with AtPIP1s was revealed, but resolution between AtPIP2 isoforms was lost (**Figure 4C**). The survivorship of AtPIP1 expressing yeast after freeze-thaw treatment were substantially lower than the AtPIP2s.

Water transport of AtPIP1s was further assessed by increasing their abundance in the PM through co-expression with *AtPIP2;5*. Yeast co-transformed with *AtPIP2;5* + *Empty* vector served as a base-level control, with less freeze-thaw tolerance than yeast carrying the *AtPIP2;5* vector alone or co-expressing two copies of *AtPIP2;5* (**Figure 4D**). This is consistent with *AtPIP2;5* + *Empty* vector yeast having reduced expression of *AtPIP2;5* as only half the plasmid load carries *AtPIP2;5*. Co-expression of *AtPIP1;1*, *1;2*, *1;3*, *1;4* or *1;5* with *AtPIP2;5* substantially improved freeze-thaw survivorship over the *AtPIP2;5* + *Empty* vector control, with AtPIP1+AtPIP2;5 co-expression increasing relative survivorship at ~40-75% compared to AtPIP2;5 alone (i.e. AtPIP2;5 + AtPIP2;5; **Figure 4D**). Co-expression revealed that all five AtPIP1 isoforms are capable of significant water transport, but they appear less effective than AtPIP2s.

Water permeability was also assessed using the traditional, but more laborious, yeast spheroplast bursting method (**Figure 4E**). During a 5-sec exposure of spheroplasts to a hypotonic solution, there is an initial phase marked by a rapid decrease in measured OD_650_ as spheroplasts swell and burst, followed by a slower reduction phase reflective of spheroplast settling. A greater value for the initial phase kinetic parameter A_1_ is indicative of more efficient water influx into yeast spheroplasts. Spheroplast bursting rates were ranked AtPIP2;7 > AtPIP2;1 > AtPIP1;5 > empty, matching the order and approximate relative differences in growth phenotypes obtained from the freeze-thaw assay. The consistency in ranking between the two methods, validated assessment of water permeability across the AtPIP family using the freeze-thaw assay which provides a platform to rapidly and comparatively evaluate relative water transport function of AQPs.

### Characterization of AtPIP H_2_O_2_ permeability

**Figure 5:**
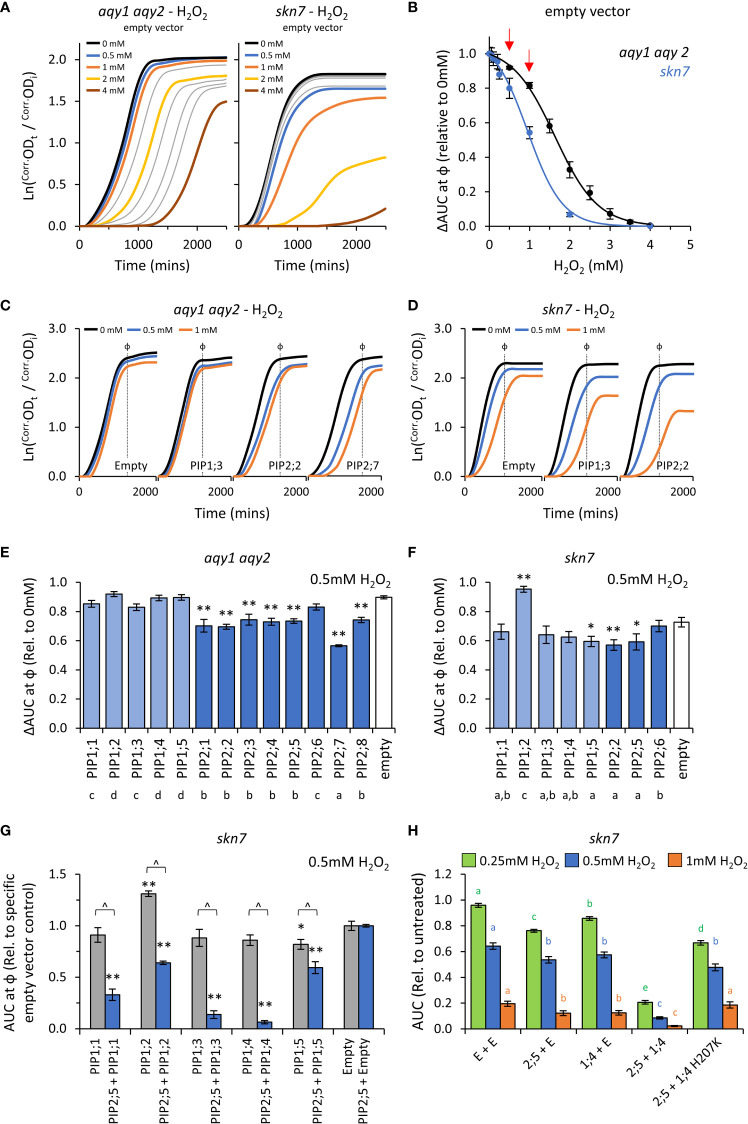
H_2_O_2_ permeability assays. **(A)**, Comparison of growth curves of two yeast strains, *aqy1 aqy2* or *skn7*, exposed to increasing H_2_O_2_ concentrations. Concentrations related to the colored lines are provided in the graph. Grey lines represent intermediate concentrations of 1.5, 2.5, 3.0, 3.5 mM, respectively for *aqy1 aqy2*, and 0.05, 0.1, 0.2, and 0.25 mM, respectively for *skn7*. **(B)**, Dose response curves showing relative AUC as a function of H_2_O_2_ concentration for each strain. s*kn7* yeast are more sensitive to H_2_O_2_ treatment than *aqy1 aqy2* yeast. Red arrows indicate H_2_O_2_ concentrations chosen for testing yeast expressing *AtPIP*. **(C)**, Example growth curves of *aqy1 aqy2* yeast expressing different *AtPIP* genes exposed to various H_2_O_2_ concentrations. **(D)**, Example growth curves of *skn7* yeast expressing different *AtPIP* genes exposed to various H_2_O_2_ concentrations. **(E)**, Relative AUC for *aqy1 aqy2* yeast expressing each AtPIP gene exposed to 0.5mM H_2_O_2_. **(F)**, Relative AUC for *skn7* yeast expressing *AtPIP1* and selected benchmark *AtPIP2* genes exposed to 0.5mM H_2_O_2_. **(G)**, Relative AUC for *skn7* yeast exposed to 0.5mM H_2_O_2_ expressing *AtPIP1* singly (grey) or together with *AtPIP2;5* (blue). Each set is standardized to their respective empty vector control. **(H)**, Relative AUC for *skn7* yeast expressing various combinations of *AtPIP* genes at 0.25, 0.5 and 1mM H_2_O_2_. All error bars are SEM. For **(E, F)**, asterisks indicate statistical difference from empty vector control, ANOVA with Fishers LSD test (* *P* < 0.05; ** *P* < 0.01); letters denote different statistical rankings across both 0.5 and 1mM H_2_O_2_, ANOVA with Tukey’s test (*P* < 0.05). For **(G)**, asterisks indicate statistical difference from empty vector control, ANOVA with Fishers LSD test (* *P* < 0.05; ** *P* < 0.01); chevrons (^) indicate statistical difference between single vs. co-expression (Student’s *t* test *P* < 0.01). For **(H)**, color coded letters denote different statistical groupings within [H_2_O_2_] treatments, ANOVA with Fishers LSD test. N = 4 bio reps for **(B)** N = 6 (2 biological reps x 3 experimental runs) for **(E)** N = 8 across 4 experimental runs for **(F)** For G, N = 12 across 6 experimental runs for single expressed AtPIPs and N = 6 across 3 experimental runs for co-expressed lines. N = 16 across 4 experimental runs for H.

The reactive oxygen species (ROS) hydrogen peroxide (H_2_O_2_), can impair yeast growth (decreasing μ and κ) and trigger cell death (prolonging λ) when internalized levels exceed the protective mechanisms of the cell (Jamieson, 1998; Madeo et al., 1999). In our setup, H_2_O_2_ treatments impaired growth of the empty vector *aqy1 aqy2* yeast (**Figure 5A**), impacting all three growth traits (λ, μ, and κ; **Supplemental Figure S5**). The effects were more prominent when using the *skn7* yeast, which is compromised in its antioxidant buffering capacity (**Figure 5A**; **Supplemental Figure S5**). 0.5mM and 1mM H_2_O_2_ were chosen as treatment concentrations as they occur at the commencement of pronounced growth inhibition (i.e. linear range of the dose response curves) (**Figure 5B**), and thus provide a greater range of detection and resolution between AQP isoforms. Testing at two concentrations (i.e. modulating diffusion potential) extends the detectable range and ability to identify weaker permeabilities and variation in the toxicity phenotypes between isoforms.

The impact on λ, μ, and κ for AtPIP-expressing yeast in response to 0.5mM and 1mM H_2_O_2_ treatment were consistent with the empty vector control exposed to increasing concentrations of H_2_O_2_ (**Figures 5A, C** and **D**). As expected, the differences between AtPIP associated H_2_O_2_ sensitivities were greater in *skn7* compared to *aqy1 aqy2* yeast (**Figures 5C, D**, and **Supplemental Figures S6A, B**).

Growth relative to the empty vector control was inhibited by 0.5mM H_2_O_2_ for all *AtPIP2* expressing *aqy1 aqy2* yeast lines except *AtPIP2;6* (**Figure 5E**). At the higher concentration (and thus diffusion potential) of 1mM H_2_O_2_, all *AtPIP2* expressing *aqy1 aqy2* yeast grew worse than empty vector control (**Supplemental Figure S6A**). Greater growth inhibition and differentiation between isoforms was observed in the more sensitive *skn7* background, with differences between select AtPIP2 isoforms (AtPIP2;2, 2;5, and 2;6) especially prominent at 1mM compared to their evaluation in the *aqy1 aqy2* background (**Supplemental Figure S6B**). The results indicated that all AtPIP2 proteins can facilitate enhanced diffusion of H_2_O_2_ across the PM to some extent, AtPIP2;6-expressing yeast was least sensitive to H_2_O_2_ treatment, while AtPIP2;7 was the most sensitive (**Figure 5E**).

*AtPIP1* expressing *aqy1 aqy2* yeast showed no indication of enhanced H_2_O_2_ uptake across the PM beyond the passive background diffusion rate, represented by the empty vector control, except for a small effect with AtPIP1;1 at 1mM H_2_O_2_ (**Supplemental Figure S6A**). When expressed in *skn7*, AtPIP1;3, 1;4 and 1;5 conferred greater sensitivity to H_2_O_2_ (at 1mM) than empty vector control, with growth reductions sitting between the most and least sensitive AtPIP2;2 and AtPIP2;6 respectively (**Supplemental Figure S6A**), indicating that these isoforms also facilitate H_2_O_2_ transport across the PM (**Supplemental Figure S6B**). Intriguingly, the *skn7* AtPIP1;2-expressing yeast grew consistently better than empty vector control (several independent transformation events, and a marginally discernable effect in the *aqy1 aqy2* background), suggesting that expression of *AtPIP1;2* alone in *skn7* somehow protects against H_2_O_2_ treatment (**Figure 5F** and **Supplemental Figure S6B**).

When AtPIP1 PM targeting was improved through co-expression with *AtPIP2;5*, all AtPIP1s dramatically increased the sensitivity of *skn7* yeast to H_2_O_2_ over the *AtPIP2;5* + *Empty* vector control. The effect was clearly evident at 0.5mM (**Figure 5G**) and even as low as 0.25mM H_2_O_2_ (**Supplemental Figure S6C**), whereas 1mM H_2_O_2_ was required to observe a significant increase in *skn7* sensitivity beyond the empty vector control when *AtPIP1*s were expressed in *skn7* yeast alone (**Supplemental Figure S6B**). Of note, the enhanced growth phenotype observed in AtPIP1;2-expressing *skn7* yeast at 1mM H_2_O_2_ was not observed when co-expressed with AtPIP2;5 (**Figure 5G**), suggesting that localization to the ER or a homotetrameric state may be required for this off-target effect. *AtPIP2;5* + *AtPIP1;3* and *AtPIP2;5* + *AtPIP1;4 skn7* lines were the most sensitive to 0.5mM H_2_O_2_, with AUC values relative to *AtPIP2;5* + *Empty* vector control of 0.1 and 0.06, respectively (**Figure 5G**). In order to confirm that co-expression of AtPIP1 was not associated with some form of hyperactivation of the AtPIP2;5 through hetero-oligomerization, we designed a mutant version of *AtPIP1;4* (*AtPIP1;4H207K*) with hindered monomeric channel activity but retained hetero-oligomerization capacity. The histidine at position 207 in AtPIP1;4 represents a strongly conserved residue located in a cytosolic loop of PIP proteins that is involved in gating of the monomeric pore (Törnroth-Horsefield et al., 2006). Mutation to a positively charged Lysine(K) mimics histidine protonation, which favors pore closure and reduces transport of substrates, including H_2_O_2_ (Tournaire-Roux et al., 2003; Verdoucq et al., 2008; Bienert et al., 2014). In an independent collection of H_2_O_2_ toxicity assays, increasing PM abundance of AtPIP1;4 through *AtPIP2;5* + *AtPIP1;4* co-expression, once again dramatically sensitized *skn7* yeast to H_2_O_2_ (**Figure 5H**). However, when *AtPIP2;5* was co-expressed with the *AtPIP1;4H207K* closed/gated mutant, the ΔAUC values resembled growth levels more similar to *AtPIP2;5 + Empty* control (**Figure 5H**). This supports the interpretation that *AtPIP1;4* was responsible for the enhanced H_2_O_2_ sensitivity of the *AtPIP2;5* + *AtPIP1;4* yeast. Collectively, the co-expression results suggest that all AtPIP1 proteins transport H_2_O_2_ and provide greater sensitivity phenotypes than AtPIP2 isoforms.

### Characterization of AtPIPs boric acid permeability

**Figure 6:**
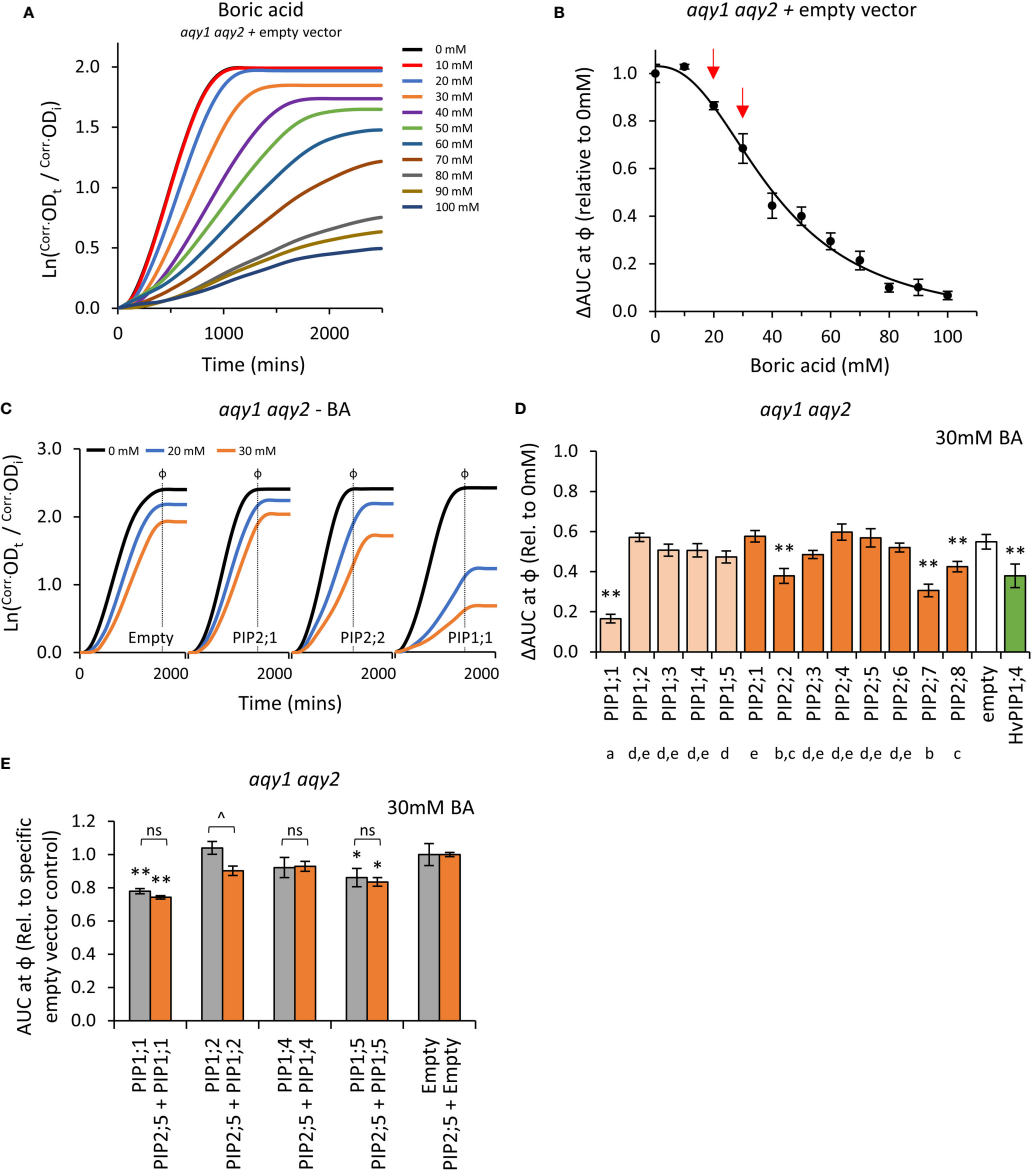
Boric acid permeability assays. **(A)**, Growth curves for *aqy1 aqy2* yeast exposed to increasing concentrations of boric acid (BA). **(B)**, Dose response curve of relative AUC as a function of boric acid concentration. Red arrows denote BA concentrations chosen for testing yeast expressing *AtPIP*. **(C)**, Example growth curves for *aqy1 aqy2* yeast expressing *AtPIP* genes exposed to 0, 20 or 30mM boric acid (BA). **(D)**, Relative AUC for *aqy1 aqy2* yeast expressing each *AtPIP* gene exposed to 30mM boric acid, with *HvPIP1;4* as a boric acid permeable control. E, Relative AUC for *aqy1 aqy2* yeast expressing *AtPIP1* singly (grey) or together with *AtPIP2;5* (orange) at 30mM boric acid. Each set is standardized to their respective empty vector control. All error bars are SEM. For **(D)**, asterisks indicate statistical difference from empty vector control, ANOVA with Fishers LSD test (* *P* < 0.05; ** *P* < 0.01); letters denote different statistical rankings across both 20 and 30mM boric acid, ANOVA with Tukey’s test (*P* < 0.05). For **(E)**, asterisks indicate statistical difference from respective empty vector control, ANOVA with Fishers LSD test (* *P* < 0.05; ** *P* < 0.01); chevrons (^) indicate statistical difference between single vs. co-expression (Student’s *t* test *P* < 0.01). For D and E, N = 6 across 3 experimental runs.

Boron is essential for yeast growth, but at high concentrations is toxic. At moderate concentrations (< 80mM) it acts as a fungistatic agent, slowing down proliferation by disrupting cell wall synthesis, but not killing the cell (Bennett et al., 1999; Schmidt et al., 2010). A range of moderate boric acid (BA; H_3_BO_3_) concentrations were tested on *aqy1 aqy2* empty vector yeast to determine treatment doses. Consistent with reports, moderate BA treatments mainly reduced the rate of growth (μ) with little impact on lag-phase (λ) (**Figure 6A**; **Supplemental Figure S7**). ΔAUC at ф relative to untreated cultures followed a single dose response curve and 20mM and 30mM BA were selected as optimal treatment concentrations (**Figure 6B**).

Changes in the growth curve characteristics (λ, μ, and κ) of AtPIP-expressing yeast in response to 20mM and 30mM BA treatment were consistent with the empty vector control exposed to increasing BA concentrations (**Figures 6A** and **C**). Five of the 13 AtPIP yeast lines were more sensitive to BA than the empty vector control (**Figures 6D** and **E**). *AtPIP1;1* expressing yeast were by far the most sensitive to BA exposure, with dramatic growth reductions even at 20mM BA (**Supplemental Figure S8**). Yeast expressing *AtPIP2;2*, *2;7* and *2;8* had BA-induced sensitivities similar to the *HvPIP1;4* positive control (Fitzpatrick and Reid, 2009). *AtPIP1;5* expressing yeast showed a small increase in BA sensitivity compared to Empty vector, which was significant in three of the four experiments (**Figure 6E**; **Supplemental Figure S8A and B**). Co-expression of *AtPIP1s* with *AtPIP2;5* did not alter BA sensitivity compared to the yeast expressing *AtPIP1*s alone at 20mM BA (**Supplemental Figure S8B**), whereas at 30mM BA, *AtPIP1;2 + AtPIP2;5* co-expression resulted in an increased BA sensitivity compared to *AtPIP1;2* alone (**Figure 6E**).

Truncation of the cytosolic N-terminal domain of PIP1, PIP2, and NIP isoforms from different plant species, has enabled boron, or similar metalloid, uptake in yeast (Bienert et al., 2008; Fitzpatrick and Reid, 2009; Kumar et al., 2014; Mosa et al., 2016). We generated and tested several PIP1 isoforms with truncations of the cytosolic N-terminal domain (*AtPIP1;2_Δ2-47_*, *AtPIP1;4_Δ2-47_* and *AtPIP1;5_Δ2-48_*). The truncated versions had similar sensitivity to BA as their full-length counterparts (**Supplemental Figure S8C**). Overall, the results indicate that five members across both the AtPIP1 and AtPIP2 sub-families are candidates for BA transport.

### Characterization of AtPIPs for urea permeability

**Figure 7:**
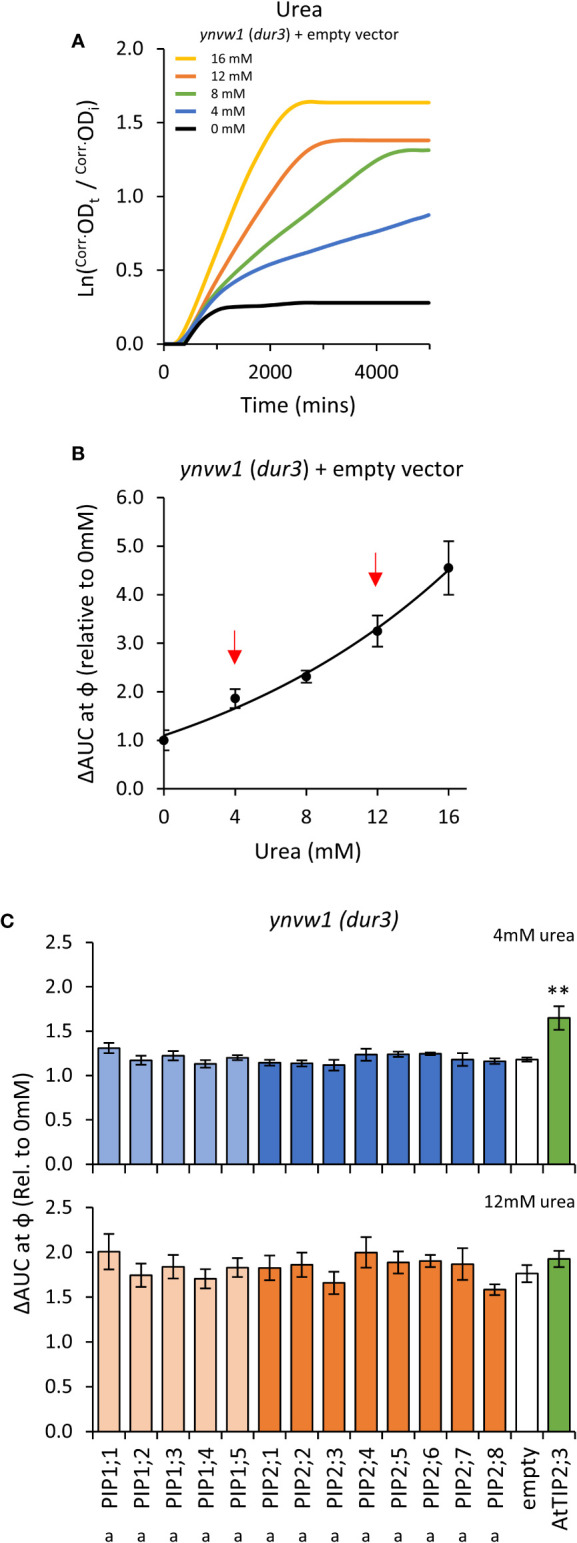
Urea permeability assays. **(A)**, Growth curves of *ynvw1* (*dur3*) yeast supplied with increasing concentrations of urea. **(B)**, Relative AUC as a function of urea concentration. Red arrows denote urea concentrations chosen for testing yeast expressing *AtPIP*. **(C)**, Relative AUC for yeast expressing each *AtPIP* grown with 4 or 12mM urea, with *AtTIP2;3* as a urea permeable control. All error bars are SEM. For **(C)**, asterisks indicate statistical difference from empty vector control, ANOVA with Fishers LSD test (** *P* < 0.01); letters denote statistical rankings across both 4 and 12mM urea, ANOVA with Tukey’s test (*P* < 0.05). For C, N = 6 across 3 experimental runs.

Growth of the empty vector *ynvw1* (*dur3*) urea uptake deficient mutant was enhanced by increasing concentrations of urea; specifically through increased maximum growth rate (μ) and carrying capacity (κ) (**Figures 7A, B**; **Supplemental Figure S9**). When urea was supplied at high concentration (i.e. high diffusion potential), all yeast lines grew similarly to the empty vector control (**Figure 7C**), indicating that the AtPIP and AtTIP2;3 (positive control) yeast cultures were healthy and capable of growing better when exposed to a urea/nitrogen concentration that imposes a higher permeability across the plasma membrane. However, in lower concentrations of urea (4 mM), none of the AtPIP expressing yeast showed improved growth compared to Empty vector, whereas the positive urea transporting control, *AtTIP2;3* (Dynowski et al., 2008a), clearly complemented the *dur3* growth phenotype (**Figure 7C**). Therefore, none of the AtPIPs appear to promote notable urea uptake.

### Characterization of AtPIPs for Na^+^ ion permeability

**Figure 8:**
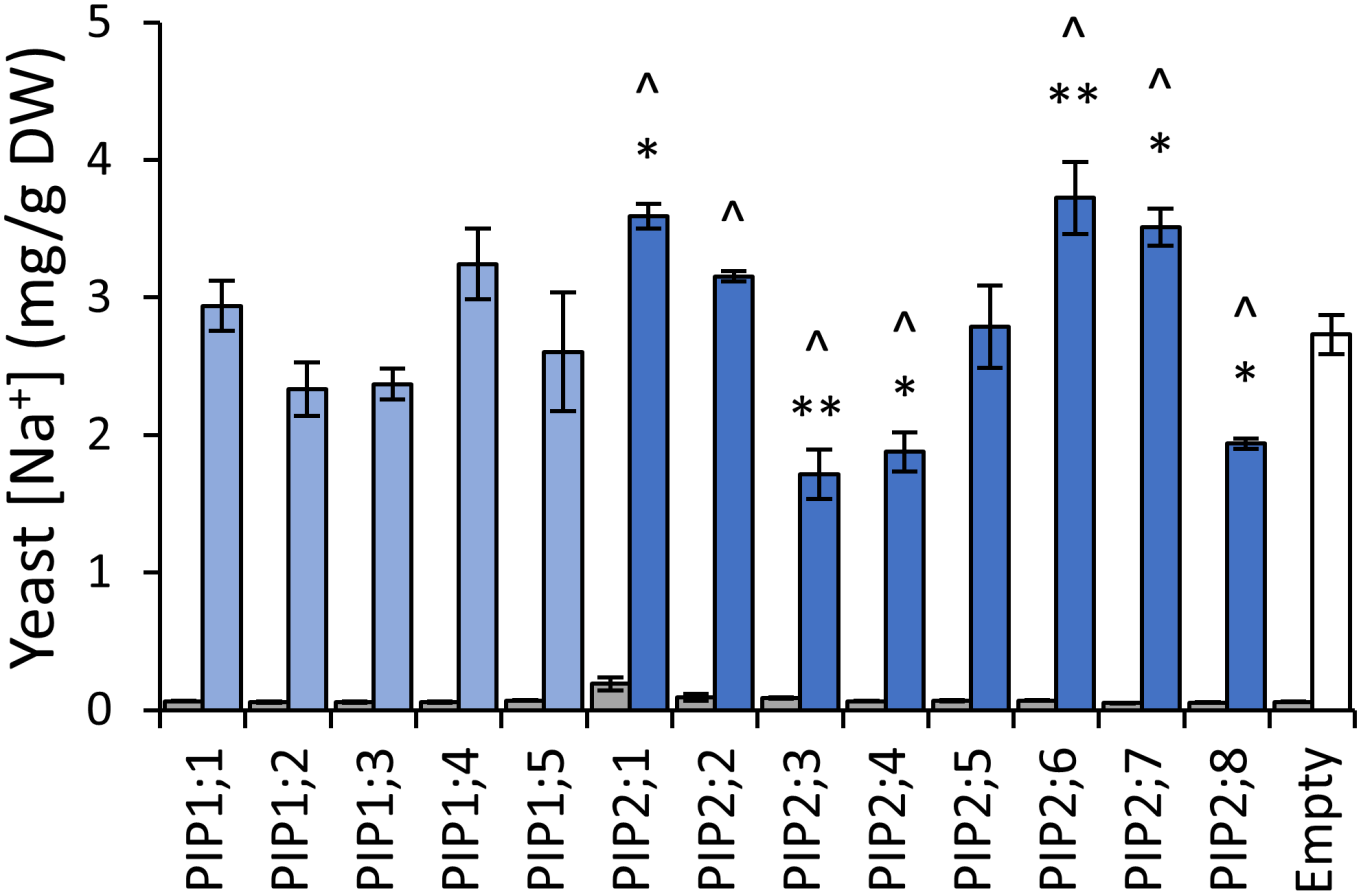
Na^+^ permeability assay. Yeast cellular sodium content before (grey) and after (blue) exposure to 70mM NaCl for 40 mins. Error bars are SEM. Asterisks indicate statistical difference from empty vector control, ANOVA with Fishers LSD test (* *P* < 0.05; ** *P* < 0.01). Chevrons (^) indicate statistical difference from empty vector control, Student’s *t* test *P* < 0.05. N = 3 for AtPIPs and N = 2 for empty vector. Two statistical tests were used to provide an added level of statistical resolution for detecting PIP-associated Na^+^ accumulation differences. The ANOVA revealed that the known NA^+^ transporter AtPIP2;1 differed to empty vector (asterisks), but the ANOVA did not detect a difference for the other known NA^+^ transporter AtPIP2;2, whereas the Student’s T-test (chevrons) did differentiate both AtPIP2;1 and AtPIP2;2 relative to empty vector. There was potential for the calcium in the Na^+^ uptake media to differentially impact Na^+^ permeability through AtPIP2;2 (Byrt et al., 2017; Kourghi et al., 2017; Qiu et al., 2020). The two statical tests did not alter interpretations of the other AtPIPs.

To assess AtPIP potential for Na^+^ transport, we quantified Na^+^ accumulation in AtPIP-expressing yeast, following short-term exposure to 70mM NaCl treatments (Qiu et al., 2020). Exposing empty vector control *aqy1 aqy2* yeast to 70mM NaCl resulted in a ~40-fold increase in the Na^+^ content relative to yeast from media without additional NaCl (**Figure 8**). The five AtPIP1 isoforms and AtPIP2;5 accumulated Na^+^ similar to the empty vector control. Yeast expressing AtPIP2;1, 2;2, 2;6 and 2;7 accumulated more Na^+^, while yeast expressing AtPIP2;3, 2;4, and 2;8 accumulated less Na^+^ than empty vector control. AtPIP2;1 served as a positive control (Byrt et al., 2017).

### The evolutionary relationship, substrate profiles, and gene expression patterns of AtPIPs

**Figure 9:**
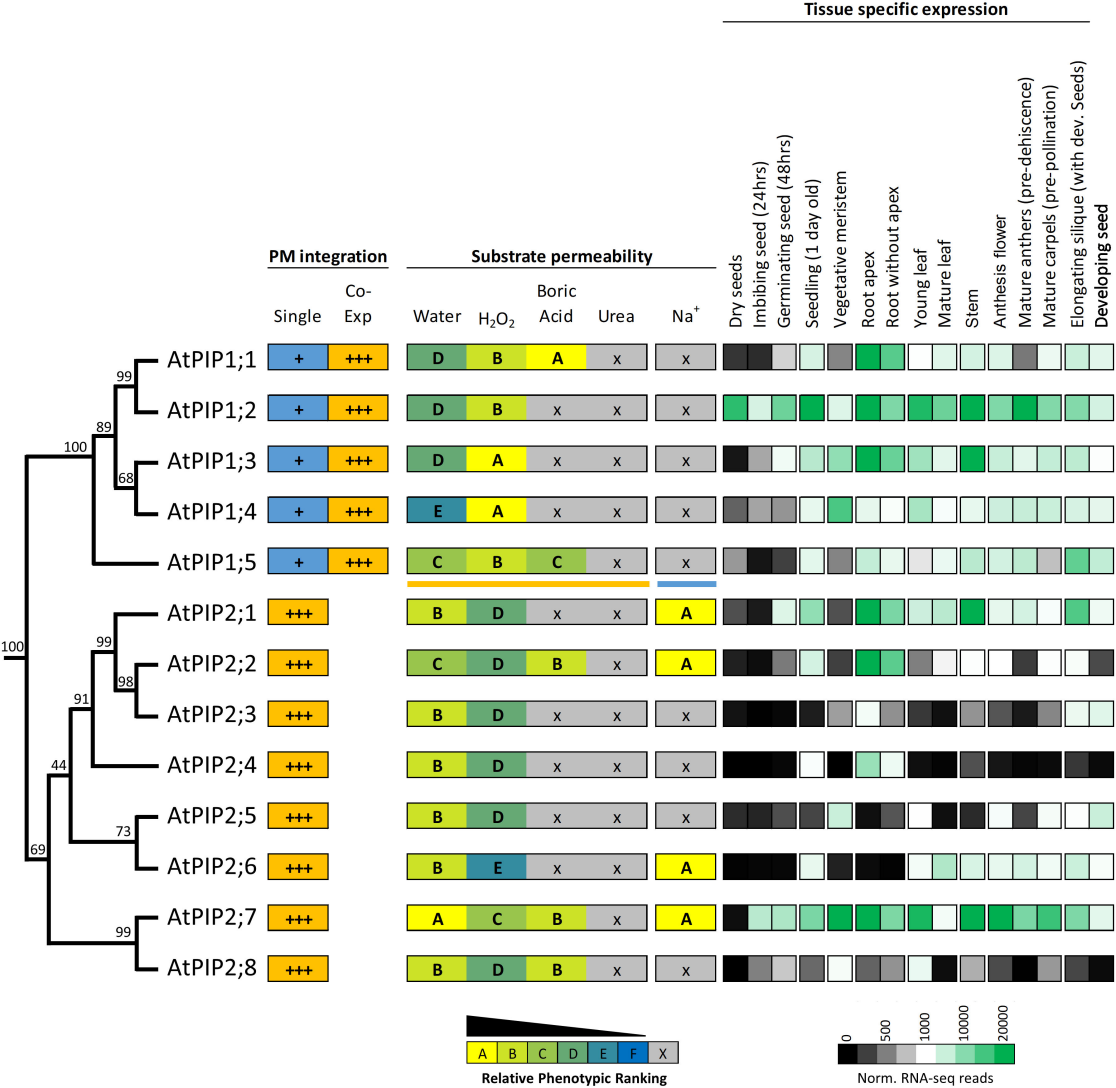
Summary of permeability and expression data for the AtPIP isoforms. The phylogenetic relationship is shown on the left, followed by strength of integration into the plasma membrane (PM) when expressed singly or co-expressed with a PIP2 (PIP1s only). Substrate permeabilities are shown in the center, and relative gene expression across different tissues during development, are shown on the right. The phylogeny is full protein sequence, using neighbor-joining method from MUSCLE alignments of protein sequences, with confidence levels (%) of branch points generated through bootstrapping analysis (n = 1000). Relative phenotype ranking for AtPIP1s are based on co-expression with AtPIP2;5 for water, H_2_O_2_, boric acid and urea (orange underline below AtPIP1;5) and singly expressed AtPIP1s for Na^+^ permeability (blue line under AtPIP1;5). Normalized tissue-specific RNA-seq data was obtained through TRAVA (http://travadb.org/) (Klepikova et al., 2016).

Protein sequence alignments reveal the high homology between AtPIPs (**Supplemental Figure S10**). Motifs associated with substrate selectivity (i.e. NPA, ar/R and Froger’s positions) are essentially identical among the AtPIPs (**Supplemental Table S2**). Gross differences are seen in the longer N-terminal and shorter C-terminal domains of AtPIP1s compared to AtPIP2s, and variation in the length of loop A (**Supplemental Table S2**). Phylogenetic analysis shows that AtPIPs divide into discrete sub-clades with distinct relationships with their substrate profiles and organ level gene expression (**Figure 9**). For example, the *AtPIP1;1* and *1;2* paralogs appear to have undergone substantial functional diversification based on their gene expression patterns. *AtPIP1;2* is the most abundantly and constitutively expressed of all *AtPIPs*, even detected at high levels in dry seed. *AtPIP1;1*, is mainly expressed in roots, being ~6-fold less prevalent in aerial tissues. This diversification in expression patterns could relate to BA transport being present in AtPIP1;1, but absent in AtPIP1;2 (**Figure 9**). The AtPIP1;3 and 1;4 paralog pair, may have evolved to transport H_2_O_2_ in preference to water given the relative phenotype rankings of higher H_2_O_2_ and lower water transport compared to other AtPIPs. Both genes are broadly expressed with largely overlapping expression domains, which together with their similar transport profiles, points towards possible functional redundancy. *AtPIP1;3* differs from *AtPIP1;4* by being more highly expressed in general, especially in the root and stem. *AtPIP1;3* expression is also up-regulated during seed imbibition and seedling germination, whereas *AtPIP1;4* is only weakly expressed at this stage of development (**Figure 9**). Intriguingly, AtPIP1;5 sits as a phylogenetic outgroup within the AtPIP1 clade, and is a candidate for water, H_2_O_2_ and BA transport. AtPIP1;5 had the highest relative phenotype ranking amongst the AtPIP1 for water transport (**Figure 9**) and *AtPIP1;5* transcripts are particularly abundant in elongating siliques and the developing seed within.

Among the AtPIP2 isoforms, AtPIP2;7 has the most diverse substrate transport profile and expression patterns, and putatively transports water, H_2_O_2_, BA, and Na^+^ ions at comparatively high efficiency based on yeast growth rankings. *AtPIP2;7* is expressed at high levels in most tissues, with the exception of mature leaves and dry seed, but is upregulated during seed imbibition and germination (**Figure 9**). Its closest relative, AtPIP2;8, is a candidate for water, H_2_O_2_, and BA transport, but *AtPIP2;8* has relatively low expression under non-stressed growth conditions (**Figure 9**). This reveals that *AtPIP2;8* is either highly cell specific, conditionally expressed, or that AtPIP2;7 is the dominant isoform of this closely related pair. The AtPIP2;5 and AtPIP2;6 phylogenetic pair are noteworthy as being apparently the least effective H_2_O_2_ transporters of all AtPIPs based on the yeast growth phenotypic rankings (**Figure 9**). *AtPIP2;5* and *AtPIP2;6* are distinctly not expressed in roots, and *AtPIP2;5* is expressed in meristematic tissue and developing seed, and *AtPIP2;6* expression is localized to aerial vegetative and reproductive tissues (**Figure 9**).

## Discussion

### High-throughput yeast micro-cultivation assays for cataloguing AQP substrate permeability profiles

Using yeast-based systems for heterologous expression and functional assessment of AQPs offers numerous advantages over other systems such as oocytes, liposomes, and artificial membranes. Key advantages include: a large range of well-characterized mutant *S. cerevisiae* strains which can be used for testing different compounds; simple monitoring of growth; scalable to high-throughput processing; and enabling power of sampling a yeast population versus single cell/event sampling in other systems.

Since liquid cultures provide improved exposure of yeast cells to substrates and enable accurate detection of smaller phenotypic growth changes relative to yeast grown on solid plates (Toussaint et al., 2006; Marešová and Sychrová, 2007; Hung et al., 2018), we developed a liquid micro-cultivation system enabling high-throughput, quantitative monitoring of yeast growth in response to treatments. The 96-well plate format offers capacity to screen multiple samples in one experiment, simplifying statistical evaluation. Optical density measurement removed the element of human subjectivity used to assess yeast spot assay phenotypes.

Although an indirect measurement, monitoring of growth inhibition/promotion in response to treatment is considered a reliable proxy for chemical uptake in yeast (Denny and Steel, 2015; Denny, 2018). This includes a growing list of yeast-based AQP studies showing that altered growth of AQP expressing yeast in response to chemical treatment reflects an enhanced intracellular accumulation of the tested substrate (Bienert et al., 2007; Bienert et al., 2008; Dynowski et al., 2008b; Fitzpatrick and Reid, 2009; Bienert et al., 2011; Kumar et al., 2014; Mao and Sun, 2015; To et al., 2015; Mosa et al., 2016; Rhee et al., 2017; Wang et al., 2019). We did not detect any indirect effects of AQP expression on yeast susceptibility to chemical treatments (**Supplemental Note 2**), and chose substrate concentrations that were at the commencement of pronounced growth effects (i.e. linear range of the dose response curves) to more ideally correlate changes in growth with improved permeability.

Curve transformation to compensate for nonlinearity in optical density measurements, provided a more accurate representation of growth parameters and culture health. The implementation of a dynamic measuring point ф, enabled standardized evaluation between different AQP-expressing yeast lines. Differential growth responses due to increased substrate diffusion into the yeast were dependably captured by the single parameter, AUC.

High AQP-abundance in heterologous systems is critical for accurate assessment of functional capacity and to avoid false-negative transport assignment (Bienert et al., 2014). Protein abundance was quantified in living yeast cells through capturing GFP fluorescence emission of C-terminal GFP translational fusions (AQP-GFP), which is an efficient high-throughput screen that correlates well with traditional Westerns (Albano et al., 1998; Ghaemmaghami et al., 2003; Drew et al., 2006; Drew et al., 2008; Scharff-Poulsen and Pedersen, 2013). High AtPIP production was achieved by careful design of our AtPIP yeast expression constructs. Protein abundance was not correlated with phenotypic responses, suggesting that the observed phenotypes were independent of protein production. The AtPIPs must also integrate into the yeast plasma membrane in order to affect substrate transport into the yeast cell. We found that AtPIP2s had strong PM integration, while AtPIP1s co-localize to the PM and ER. Poor PM localization of PIP1s expressed alone in heterologous systems is a common phenomenon (Yaneff et al., 2015), likely due to sequence differences in the diacidic motif, LxxxA and C-terminal phosphorylation protein motifs known to control PIP2 PM trafficking (**Supplemental Table 2**) (Chevalier and Chaumont, 2015). The exact composition of diacidic and LxxxA motifs vary, particularly between the phylogenetically distinct [2;1, 2;2, 2;3, 2;4] and [2;5, 2;6, 2;7, 2;8] groups (**Supplemental Table 2**), yet all AtPIP2s localized efficiently to the yeast PM. In plants, the phylogenetically distinct AtPIP2;1 and AtPIP2;7 also localize efficiently to the PM (Sorieul et al., 2011; Hachez et al., 2014). This reveals flexibility in these motif sequences that must work together with other domains (e.g. TMH2; Wang et al., 2019) to control ER to PM trafficking. PIP2 proteins can physically interact with PIP1s and facilitate PM integration in both host and heterologous systems (Jozefkowicz et al., 2017). We enhanced AtPIP1 PM localization by co-expression with AtPIP2;5, thereby enabling a more robust assessment of substrate permeability between the two PIP sub-types. Protein levels and PM targeting efficiency (e.g. using co-expression) were comparable between the AtPIPs, meaning any differences in permeability profiles were independent of these factors and may be attributed to the intrinsic differences in the sequences of the AtPIPs.

### AtPIP water permeability

Water permeability is the most extensively studied AQP function across species. Most AtPIPs have been confirmed to transport water (AtPIP1;1, 1;2, 1;3, 2;1, 2;2, 2;3, 2;4, 2;6, and 2;7) (Kammerloher et al., 1994; Tournaire-Roux et al., 2003; Heckwolf et al., 2011; Byrt et al., 2017; Kourghi et al., 2017; Wang et al., 2020a). These assessments are from different studies and systems making it difficult to directly compare relative transport ranking. Here, water permeability was assessed for the complete set of AtPIPs using a freeze-thaw assay that we refined for rapidly evaluating water transport capacity of AQPs. We found that all AtPIP isoforms were capable of water transport, with AtPIP2s having higher apparent water transport capacity than AtPIP1s, even when PIP1s are efficiently targeted to the PM through co-expression with AtPIP2;5. Past studies concluding that PIP1s have low/no permeability to water, likely reflect the inefficient PM targeting of PIP1s when expressed alone in heterologous systems, hiding their true water transport ability [reviewed in (Yaneff et al., 2015)].

PIPs provide a transcellular route for water flow in the plant, from water uptake by roots to transpiration loss from aerial tissues (Groszmann et al., 2017). Both AtPIP1 and AtPIP2 isoforms play major roles in water flow in Arabidopsis (Javot et al., 2003; Prado et al., 2013; Sade et al., 2014). Overlapping expression patterns suggest substantial functional redundancy, which limits the ability of reverse genetic studies to resolve the contribution of each AtPIP to water flow. For example, single loss-of-function mutants of high leaf-expressing isoforms *Atpip1;2*, *Atpip2;1* and *Atpip2;6* each show a ~20% reduction in rosette hydraulic conductivity, which worsens to ~39% in the triple mutant (Prado et al., 2013). Our observations that AtPIP2;7 has high apparent water permeability and its transcripts are abundantly expressed in developing leaves (**Figure 9**), suggests it may also contribute to rosette hydraulic conductivity. Similarly, redundancy for root hydraulic conductance is likely given that the 10-20% reductions seen in single *Atpip* mutants falls short of the ~64% decrease achieved using AQP chemical blockers (Maurel et al., 2015). Four of the seven *AtPIP*s abundantly expressed in roots (**Figure 9**), had high apparent water permeability (AtPIP2;1, 2;2, 2;4, 2;7) and are strong candidates for multiple knock-out mutant studies.

More intricate developmental processes relying on cell-to-cell water movement through AtPIPs are emerging. For example, guard cell closure (Grondin et al., 2015), lateral root emergence (Péret et al., 2012), and pollen germination on stigmatic papillae (Windari et al., 2021). A number of AtPIPs are expressed in the flower, developing silique and seeds. In these tissues, AtPIP water transport could have roles in petal expansion, anther and pollen development, and assist in the supply of nutrients to the developing seed as seen in other species (Wang et al., 2020b; Hoai et al., 2020).

### AtPIP H_2_O_2_ permeability

When expressed in *aqy1 aqy2* and/or *skn7* yeast, all AtPIP2s and AtPIP1s (co-expressed with AtPIP2;5) led to enhanced toxicity phenotypes indicative of H_2_O_2_ transport in yeast (**Figure 5**), which is consistent with the similar physicochemical properties of H_2_O_2_ and water (Almasalmeh et al., 2014). AtPIP1 expression alone did not lead to increased yeast cell sensitivity to H_2_O_2_ exposure but an unexpected ‘protective’ growth phenotype in AtPIP1;2 yeast lines was observed. Previous growth-based assessments with yeast did not assign H_2_O_2_ permeability to AtPIP1 isoforms and showed mixed results for AtPIP2 isoforms (Hooijmaijers et al., 2012; Wang et al., 2019; Wang et al., 2020a). Variation in previously observed growth-based assessments may have been due to inadequate protein production, insufficient PM targeting (as shown in our experiments), choice of yeast strain, sub-optimal H_2_O_2_ concentrations, or use of solid medium spot growth assays.

The potential for H_2_O_2_ transport through AtPIP1s was recently hinted at using AtPIP1/2 chimeric proteins that more effectively localize to the PM (Wang et al., 2019). However, in addition to harboring PM targeting motifs, the substituted PIP2 domains also contribute to the pore lining, making it uncertain how representative these chimeric proteins are of native AtPIP1 function. In our system, we found that native AtPIP1 proteins were indeed capable of transporting H_2_O_2_, and when efficiently targeted to the PM through co-expression with AtPIP2;5, appeared more effective at transporting H_2_O_2_ than AtPIP2 isoforms.

H_2_O_2_ is an indispensable signaling molecule involved in many aspects of plant growth, biotic defense and abiotic stress responses, reliant on AQPs to facilitate its movement between sub-cellular compartments and cells (Černý et al., 2018; Fichman et al., 2021). The diversity of AtPIP expression patterns *in planta* and AtPIP ability to facilitate H_2_O_2_ transport, may enable fine tuning of H_2_O_2_ signaling. Direct physiological evidence in Arabidopsis is emerging, with H_2_O_2_ transport through AtPIP2;1 involved in triggering stomatal closure (Rodrigues et al., 2017) and mediating systemic acquired acclimation to abiotic stress (Fichman et al., 2021), and AtPIP1;4 mediating H_2_O_2_ triggered immunity against pathogen attack (Tian et al., 2016). Our results showing the extreme H_2_O_2_ sensitivity conferred by expression of *AtPIP1;3/1;4* paralogs in *skn7* yeast suggests these paralogous AtPIPs have evolved high apparent H_2_O_2_ transport capacity with largely overlapping tissue-specific expression patterns *in planta*. This functional and tissue expression redundancy suggests that AtPIP1;3 could also mediate H_2_O_2_ signaling for plant immunity. Supporting this idea, H_2_O_2_ translocation into the cell is decreased but not eliminated in the *atpip1;4* single mutant (Tian et al., 2016); and only *AtPIP1;4* and *AtPIP1;3* are rapidly up-regulated in response to H_2_O_2_ treatment of leaves (Hooijmaijers et al., 2012). The latter would be a consistent response to the apoplastic H_2_O_2_ produced upon pathogen recognition and facilitating its entry into the cell to trigger immune responses (Tian et al., 2016). *AtPIP1;3* transcripts are not present in dry seed, but are substantially induced during seed imbibition and germination. Hydrating seed releases H_2_O_2_ as a signal to promote germination, and may involve AtPIP1;3, which would be consistent with the involvement of AQPs in the germination process (Hoai et al., 2020). Further investigation into a role for AtPIP1;3 in plant immunity and seed germination appears warranted.

### AtPIP boric acid permeability

Five AtPIPs increased yeast sensitivity to BA exposure, indicating transport of this substrate. The relative phenotypic rankings of most to least sensitive was AtPIP1;1 > AtPIP2;2 = AtPIP2;7 = AtPIP2;8 > AtPIP1;5. AQP-mediated BA transport is generally associated with NIP-type AQPs (Pommerrenig et al., 2015). However, a growing number of PIP isoforms from different species are being found capable of transporting BA in heterologous systems; ZmPIP1;1 (Dordas et al., 2000), OsPIP1;3 and OsPIP2;6 (Mosa et al., 2016), OsPIP2;4 and OsPIP2;7 (Kumar et al., 2014), and HvPIP1;3 and HvPIP1;4 (Fitzpatrick and Reid, 2009).

The AtPIPs identified in this study as candidates for BA transport, are expressed in all tissue types *in planta* and may help coordinate uptake and distribution of this essential micronutrient, and provide tolerance *via* efflux under toxic concentrations. Interestingly, AtPIP1;1 increased yeast sensitivity to BA, but its paralog AtPIP1;2 did not. In Arabidopsis, *AtPIP1;1* expression is unaltered in roots and minimally in shoots under toxic boron conditions, whereas *AtPIP1;2* is substantially repressed (Macho-Rivero et al., 2018). This suggests AtPIP1;1 may have undergone substantial functional diversification since duplication with *AtPIP1;2. AtPIP1;2* is widely and highly expressed throughout all tissues and facilitates CO_2_ diffusion into chloroplasts for photosynthesis (Heckwolf et al., 2011), whereas we suggest AtPIP1;1 may be specialized for micronutrient uptake from the soil.

Although a native physiological role for PIP boron transport is not yet confirmed in any species, improved tolerance to boron toxicity in Arabidopsis over-expressing boron permeable rice PIPs, points towards a possible role (Kumar et al., 2014; Mosa et al., 2016).

### AtPIP urea permeability

Urea differs massively from water with respect to size, polarity, and other physicochemical properties. When expressed in the *dur3* yeast strain, none of the AtPIPs improved yeast growth like that seen for the urea permeable positive control AtTIP2;3, suggesting no AtPIP was capable of permeating urea. This is consistent with urea being too large to pass through the narrow aperture of the AtPIP a/R filter (**Supplemental Table S2**) (Dynowski et al., 2008a; Dynowski et al., 2008b).

### AtPIP Na^+^ permeability

Yeast tolerance of salt toxicity is associated with osmo-resistance (Stratford et al., 2019), meaning that AtPIP water transport could confound growth data for AtPIP expressing yeast grown at high salt concentrations. Therefore, assessment of AtPIP Na^+^ permeability from yeast growth requires a tailored mutant (Sychrova, 2004). Instead, to screen for AtPIP Na^+^ transport, we quantified intracellular yeast Na^+^ content directly. We confirmed previous reports of Na^+^ permeability for AtPIP2;1 and AtPIP2;2 (Byrt et al., 2017; Qiu et al., 2020), and observed that AtPIP2;6 and AtPIP2;7 also appear permeable to Na^+^. The latter is at odds with previous electrophysiological experiments on *AtPIP2;7* expressing oocytes that report AtPIP2;7 is not permeable to Na^+^ (Kourghi et al., 2017). The contrasting findings could reflect different heterologous expression systems and detection techniques, but investigation of post-translational regulation of AtPIP2;7 function is warranted since phosphorylation of the AtPIP2;1 and HvPIP2;8 C-terminal domains have been shown to influence ion permeability (Qiu et al., 2020; Tran et al., 2020).

We observed no enhanced Na^+^ accumulation in yeast expressing AtPIP1s alone. Since the central pore, formed in the middle of tetrameric AQP complex, is the proposed pathway for monovalent ions (Yool and Weinstein, 2002), we did not screen yeast co-expressing AtPIP1s with AtPIP2;5. This would change the structure of the central pore and make interpretation of results ambiguous, as seen for CO_2_ and Na^+^ transport through the central pore of PIP hetero-tetramers (Otto et al., 2010; Byrt et al., 2017).

The dual permeability to water and solutes of certain AtPIPs may help build high turgor during cell expansion. For example, AtPIP2;1 is involved in lateral root emergence where the primordia pushes through the overlying tissues (Péret et al., 2012). Our observations that AtPIP2;7 has dual water and solute transport capacity and is upregulated during seed imbibition and germination, implies a role aiding the massive influx of water needed for the radicle to puncture through the seed coat. Moreover, expression of *AtPIP2;7* in seeds responds to two antagonistically acting phytohormones (GA and ABA) that regulate seed dormancy versus germination (Hoai et al., 2020).

### Why the differences in growth and toxicity phenotypes between AtPIP isoforms?

We observed differences among AtPIPs in their relative phenotypic rankings when tested for water, H_2_O_2,_ BA or Na^+^ transport. This is puzzling given the near identical residue signatures of motifs classically considered to govern substrate selectivity (i.e. NPA, ar/R, and Froger’s positions) (**Supplemental Table S2**; **Figure S10**), and indicates the involvement of other domains yet to be defined. Variation in transport efficiency for water and H_2_O_2_ is likely to be associated with subtle differences in residues forming the monomeric pore that alter the number of hydrogen bonds with the substrate, or that shift, even slightly, the spatial configuration of the pore diameter (Horner et al., 2015; Mom et al., 2021). Differences in the sensitivity of gating regulation and the degree of ‘openness’ or ‘open probability’ is another possible factor not only for regulating the capacity for transport but also switching between substrate preferences possibly through shifting between monomeric versus central pores (Kourghi et al., 2017; Vitali et al., 2019; Qiu et al., 2020; Lu et al., 2022).

Increased Na^+^ accumulation was only detected for some AtPIPs, pointing to differences in central pore features (Yool and Weinstein, 2002). The route for BA through PIPs is unknown, but mutant analysis suggests the monomeric pore is most likely (Dynowski et al., 2008b). However, we cannot exclude the central pore given its hydrophobic profile and hypothesized ability to open wider through helix rotation (Tyerman et al., 2021). Structural changes to the central pore of hetero-tetramers would also account for the inability to improve AtPIP1;1 and AtPIP1;5 boric acid permeability when co-expressed with AtPIP2;5.

The limited sequence differences between the AtPIPs (**Supplemental Figure S10**), should make identification of substrate specificity residues easier and feasible to explore through mutation approaches.

## Conclusion

AQPs are membrane proteins with wide-ranging transport capabilities, for which deciphering functional determinants will be essential for their successful deployment in industrial and crop biotechnological applications. Building a substantial catalogue of transport profiles for AQPs is needed for association studies to identify residues of functional relevance, and for the development of an AQP core differential set that would enable effective screening of new and novel substrate permeabilities. The testing framework described in this study enables efficient cataloguing of putative transport functionality of diverse AQPs. We applied this framework to produce comparative substrate transport profiles for water, hydrogen peroxide, boric acid and urea across the entire AtPIP subfamily. Na^+^ transport was assessed using elemental analysis techniques. Our results indicate that all AtPIPs facilitated water and H_2_O_2_ transport, although their growth phenotypes varied, and none were candidates for urea transport. For BA and Na^+^ transport, AtPIP2;2 and AtPIP2;7 were the top candidates, with yeast expressing these isoforms having the most pronounced toxicity response to BA exposure and accumulating the highest amounts of Na^+^. Such data is critical towards informing structure-function relationships and being able to develop designer AQPs with tailored functionality. Until now, an inability to robustly phenotype at scale was the key constraint in producing a sufficiently large catalogue of AQP transport profiles. However, the high-throughput yeast-based phenotyping framework that we have reported here, provides a sufficient solution to this previous bottleneck. This framework could in future be applied to test for solute transport for isoforms in other AQP subfamilies from diverse species, and the principles of the individual assays could be further adapted to test additional substrates. Even just among the 13 AtPIPs, we observed distinct substrate profiles, that aligned with evolutionary relatedness and known biological functions in Arabidopsis. These functional differences are particularly intriguing given the near identical residues in motifs classically considered to govern substrate selectivity (i.e. NPA, ar/R and Froger’s positions). This data suggests involvement of other, yet to be determined, domains influencing AQP substrate permeability, that should be revealed with the expanded application of this phenotyping framework and generation of the transport profile catalogue.

## Data availability statement

The original contributions presented in the study are included in the article/**Supplementary Material**. Further inquiries can be directed to the corresponding authors.

## Author contributions

MG conceived the original screening, framework, future applications, and research plans, and made the yeast expressing the AtPIP constructs. MG and AD developed the micro-cultivation methodology, established optimal treatment concentrations and wrote the manuscript. MG developed data processing methodology, MG performed the AtPIP yeast screening experiments and analysis. MG and WC performed AtPIP interaction and yeast spheroplast analysis. JQ and SM developed and performed the sodium uptake assay with supervision by CB. MG, JE, CB, AD and SM analyzed the data. All authors critically reviewed and edited the manuscript. MG and AD agree to serve as the authors responsible for contact and ensure communication. All authors contributed to the article and approved the submitted version.
