# Supplementary material for: A high-throughput yeast approach to characterize aquaporin permeabilities: Profiling the Arabidopsis PIP aquaporin sub-family
Source: Front Plant Sci. 2023 Jan 19;14:1078220. doi: 10.3389/fpls.2023.1078220 (PMC9907170; doi:10.3389/fpls.2023.1078220)
Supplement: Supplementary file 2 [file DataSheet_2.pdf]

Supplemental Figures

Permeability profiling of all 13 Arabidopsis PIP aquaporin genes using a high throughput yeast approach

Michael Groszmann[1], Annamaria De Rosa[1], Weihua Chen[1], Jiaen Qiu[2], Samantha A McGaughey[1], Caitlin S. Byrt[1] and John R Evans[1]

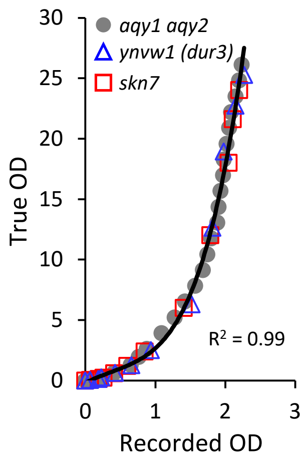

Supplemental Figure S1. Adjusting for non-linearity of OD measurements at high cell density. Correlative analysis between 'recorded' OD on plate reader versus 'true' OD as determined through dilution factors. The micro-volume data was fitted with a polynomial function ($y = 1.9481x^4 - 4.2474x^3 + 5.0329x^2 + 0.3441x$), that converts 'recorded' OD_{650} at 200ul readings to 'true' OD_{650} readings at 1cm pathlength.

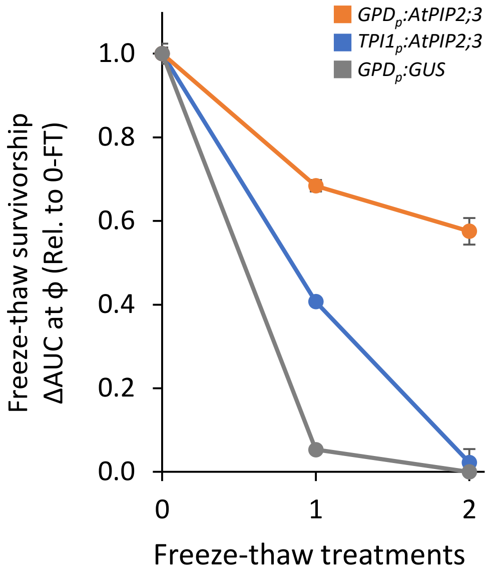

Supplemental Figure S2. The highly active *GPD* promoter confers greater AQP enhanced water permeability over the less active *TPI1* promoter. *aqy1 aqy2* double mutant yeast carrying GPD_p:AtPIP2;3 show superior rates of freeze-thaw survivorship (a proxy for water permeability) over yeast carrying TPI_p:AtPIP2;3. The *GPD* (*TDH3*) promoter is reported to be approximately twice as active as the *TPI1* promoter in the glucose-rich conditions of our micro-cultures (Partow *et al.*, 2010, Peng *et al.*, 2015). This substantial difference in phenotype, highlights not only the benefit of using the strong GPD promoter, but also the importance of having high AQP production to ensure robust phenotypes for evaluation. Freeze-thaw survivorship was determined by the cumulative growth (AUC) from culture inception to time-point ϕ between untreated and freeze-thawed cells. Yeast expressing the β-glucuronidase reporter gene driven by the GPD promoter was used as negative *AQP* control. Mean ± SEM, n = 3 biological replicates. *GPD - GLYCERALDEHYDE-3-PHOSPHATE DEHYDROGENASE (GAPDH) ISOZYME 3. TPI - TRIOSE PHOSPHATE ISOMERASE*

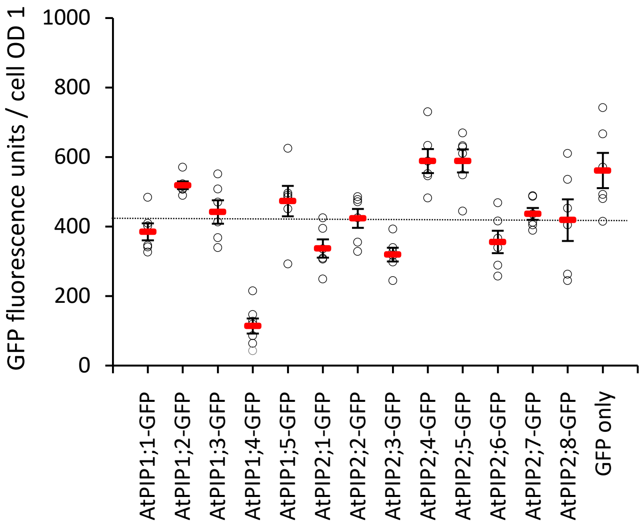

Supplemental Figure S3. Quantification of AtPIP protein abundance in intact yeast. GFP fluorescence intensity of yeast cultures expressing AtPIP:GFP fusion proteins were used to assess heterologous AtPIP protein production between the various lines. Assay was performed in the *aqy1 aqy2* null mutant yeast strain. Dotted line is the average GFP intensity signal across all AtPIPs. GFP fluorescence intensity standardised to a cell $^{Corr.}OD_{660}$ = 1. N = 6 biological replicates across 6 experimental runs, each measured in duplicate across three dilutions (see supplemental materials and methods).

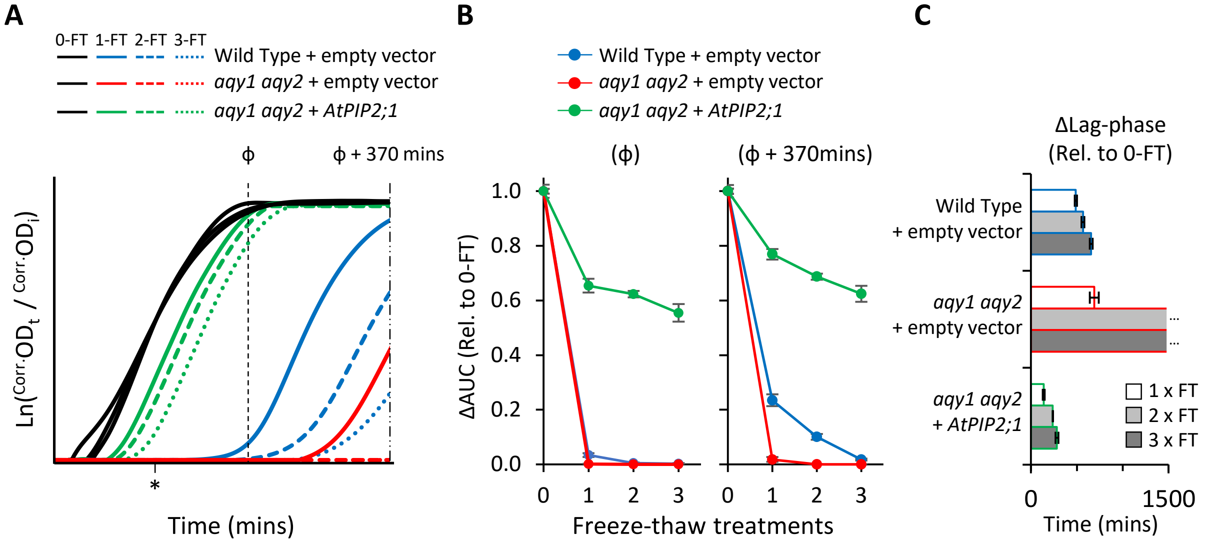

Supplemental Figure S4. Establishing the freeze-thaw assay for water permeability. A, Growth curves of WT, *aqy1 aqy2*, and *aqy1 aqy2* expressing *AtPIP2;1* yeast after 0, 1, 2, or 3 freeze-thaw (FT) treatments. For illustrative purposes the untreated curves have been standardized to a fixed lag-point (*) and the treated curves remain relative to their respective control. **B**, ΔAUC values at two different measuring points, ϕ and ϕ + 370 mins, demonstrating that the *aqy1 aqy2* yeast are more sensitive than the WT yeast to freeze-thaw treatment with an incremental decrease in ΔAUC after successive freeze-thaw treatments. Expression of the water permeable *AtPIP2;1* in the *aqy1 aqy2* yeast confers a significantly higher survival rate. **C**, Freeze-thawing prolongs the lag phase, which is consistent with a reduction in the viable cell count of the starting population due to the freeze-thaw treatments.

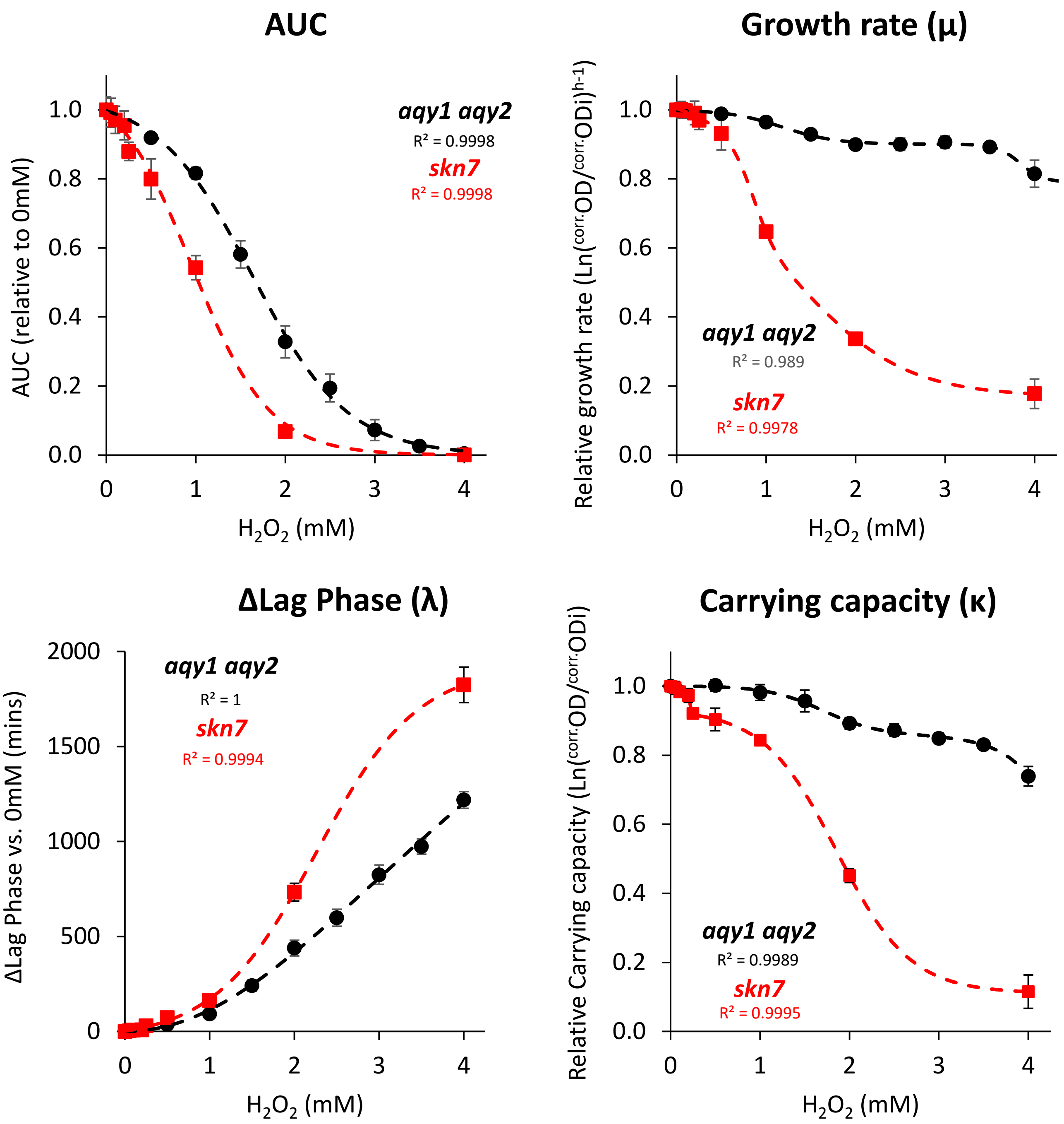

Dose Response curve:

$$y = A1 + \frac{A2 - A1}{1 + 10^{(LOGx0 - x)p}}$$

Bi-Dose Response curve:

$$y = A1 + (A2 - A1)\left[\frac{p}{1 + 10^{(LOGx01 - x)h1}} + \frac{1 - p}{1 + 10^{(LOGx02 - x)h2}}\right]$$

Supplemental Figure S5. Calibrating H_2O_2 treatments for our yeast growth assay. Growth responses of *aqy1 aqy2* and *skn7* yeast carrying empty vector control, to increasing concentrations of H_2O_2 added to the growth medium. As expected, the *skn7* mutant yeast strain, which is compromised in aspects of its antioxidant buffering capacity, was substantially more sensitive to H_2O_2 treatment than *aqy1 aqy2*. Both yeast strains showed phasic responses in μ and κ, indicating points where H_2O_2 concentrations overwhelm ROS buffering mechanisms. The growth responses captured by AUC and were fitted by a single dose response curve. Curve fitting of individual traits: μ - Bi-Dose response curve; λ - Dose response curve; κ - Bi-Dose response curve. R^2 values for each curve are listed on each graph

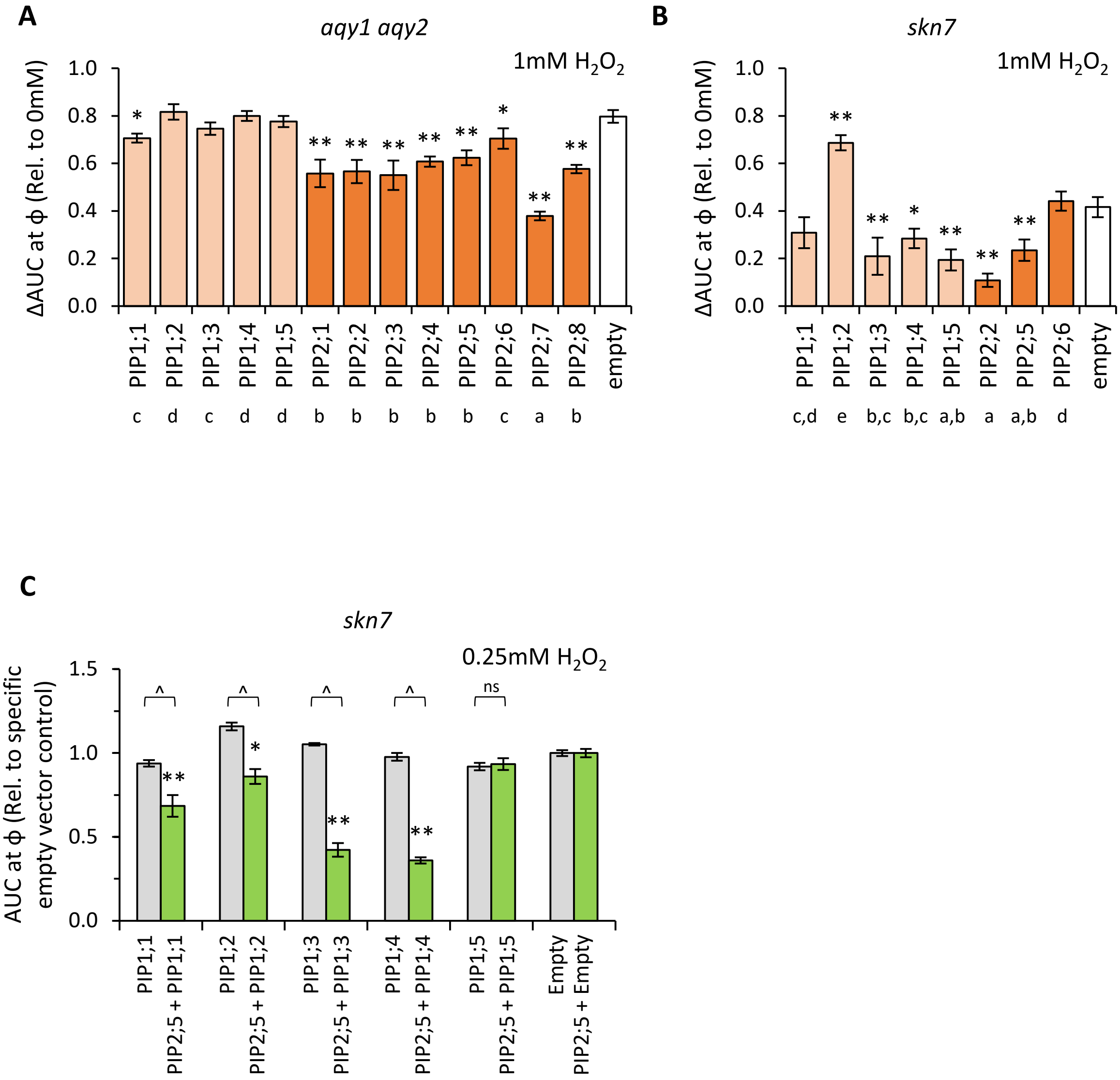

Supplemental Figure S6. H_2O_2 permeability assays. A, Relative AUC for *aqy1 aqy2* yeast expressing each AtPIP gene exposed to 1mM H_2O_2. **B**, Relative AUC for *skn7* yeast expressing *AtPIP* genes exposed to 1mM H_2O_2. **C**, Relative AUC for *skn7* yeast exposed to 0.25mM H_2O_2 expressing *AtPIP1* singly (grey) or together with *AtPIP2;5* (green). Each set is standardized to their respective empty vector control. All error bars are SEM. For A and B, asterisks indicate statistical difference from empty vector control, ANOVA with Fishers LSD test (* $P < 0.05$; ** $P < 0.01$). For C, asterisks indicate statistical difference from respective empty vector control, ANOVA with Fishers LSD test (* $P < 0.05$; ** $P < 0.01$); chevrons (^) indicate statistical difference between single vs. co-expression (Student's t test $P < 0.01$). N = 6 across 3 experimental runs for A. N = 8 across 4 experimental runs for B. For C, N = 12 across 6 experimental runs for single expressed AtPIPs and N = 6 across 3 experimental runs for co-expressed lines.

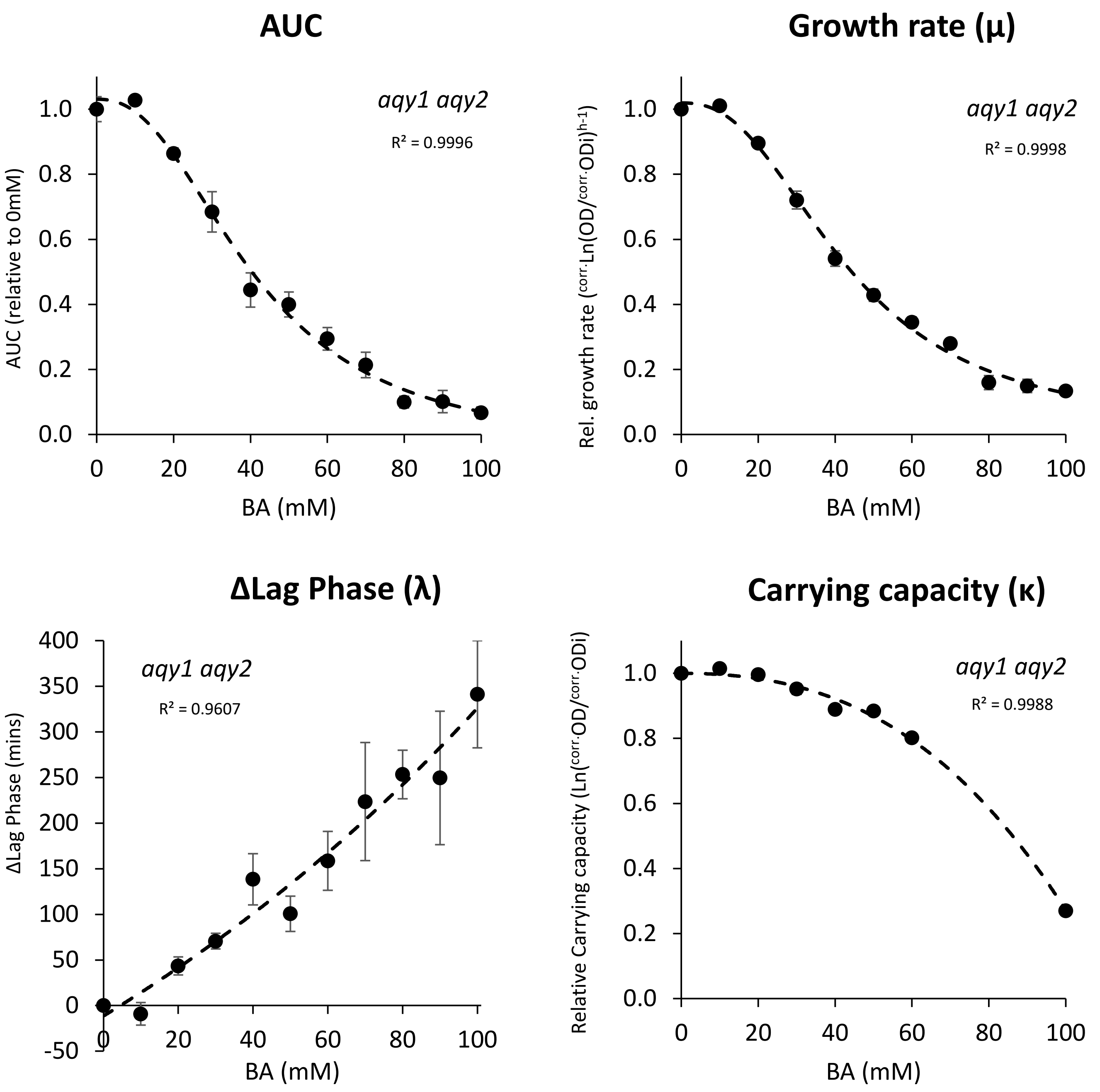

Logistic curve: $y = \frac{A_1 - A_2}{1 + (x / x_0)^p} + A_2$ Cubic curve: $y = A + Bx + Cx^2 + Dx^3$

Supplemental Figure S7. Calibrating boric acid treatments for our yeast growth assay. Growth responses of *aqy1 aqy2* yeast carrying empty vector control to increasing concentrations of boric acid (BA) added to the growth medium. BA treatments mainly reduced the rate of growth (μ). The growth responses captured by AUC was best fitted with a logistics curve. Curve fitting of individual traits: μ - logistic curve; λ – logistic curve; κ - cubic curve. R^2 values for each curve are listed on each graph

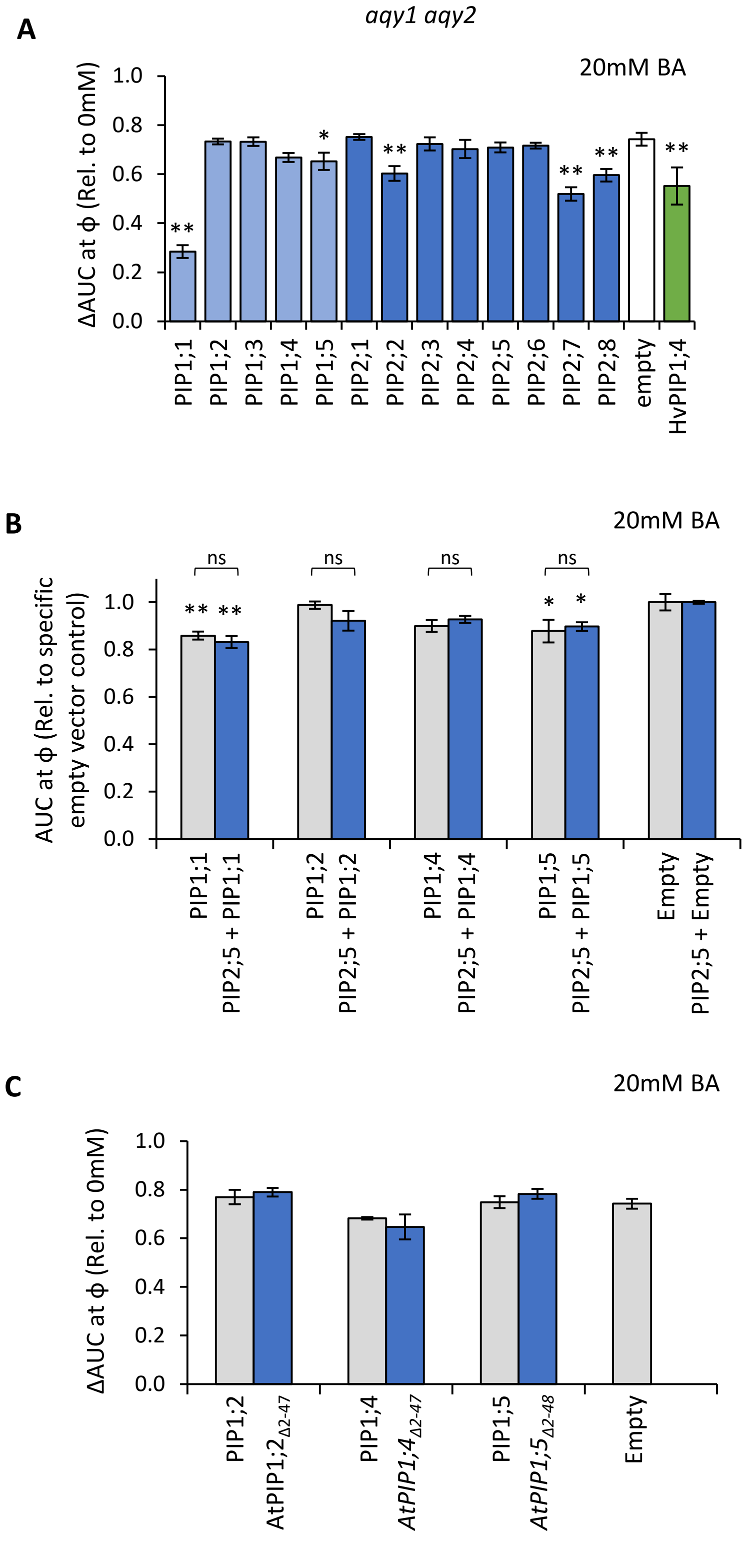

Supplemental Figure S8. Boric acid permeability assays. A, Relative AUC for *aqy1 aqy2* yeast expressing each *AtPIP* gene exposed to 20mM boric acid, with *HvPIP1;4* as a boric acid permeable control. **B**, Relative AUC for *aqy1 aqy2* yeast expressing *AtPIP1* singly (grey) or together with *AtPIP2;5* (blue) at 20mM boric acid. Each set is standardized to their respective empty vector control. **C**, Relative AUC for *aqy1 aqy2* yeast expressing either the full length or N-terminal deleted versions of listed AtPIP1s. All error bars are SEM. For A, asterisks indicate statistical difference from empty vector control, ANOVA with Fishers LSD test (* $P < 0.05$; ** $P < 0.01$). For B, asterisks indicate statistical difference from respective empty vector control, ANOVA with Fishers LSD test (* $P < 0.05$; ** $P < 0.01$. For A and B, N = 6 across 3 experimental runs. For C, N = 4 across 2 experimental runs.

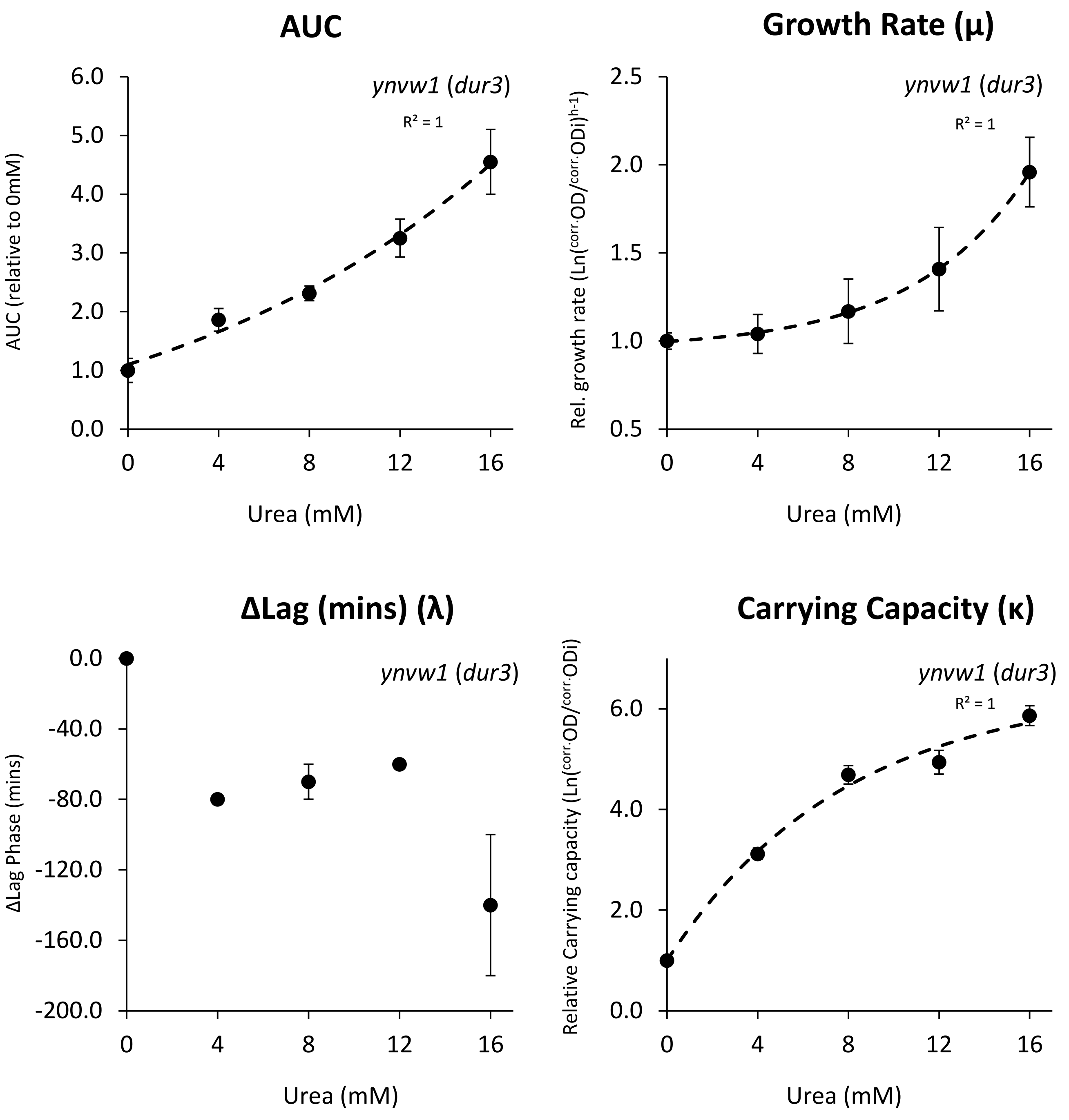

Exponential curve: $y = y_0 + Ae^{R_0 x}$

Supplemental Figure S9. Calibrating urea treatments for yeast growth assay. Growth responses of *ynvw1* (*dur3*) yeast carrying empty vector control to increasing concentrations of urea added to the otherwise nitrogen free growth medium. Urea supplementation mainly improves µ and κ. The growth response captured by AUC was best fitted with a exponential curve. Curve fitting of individual traits: µ - exponential curve; λ – no fit; κ - exponential curve. R^2 values for each curve are listed on each graph

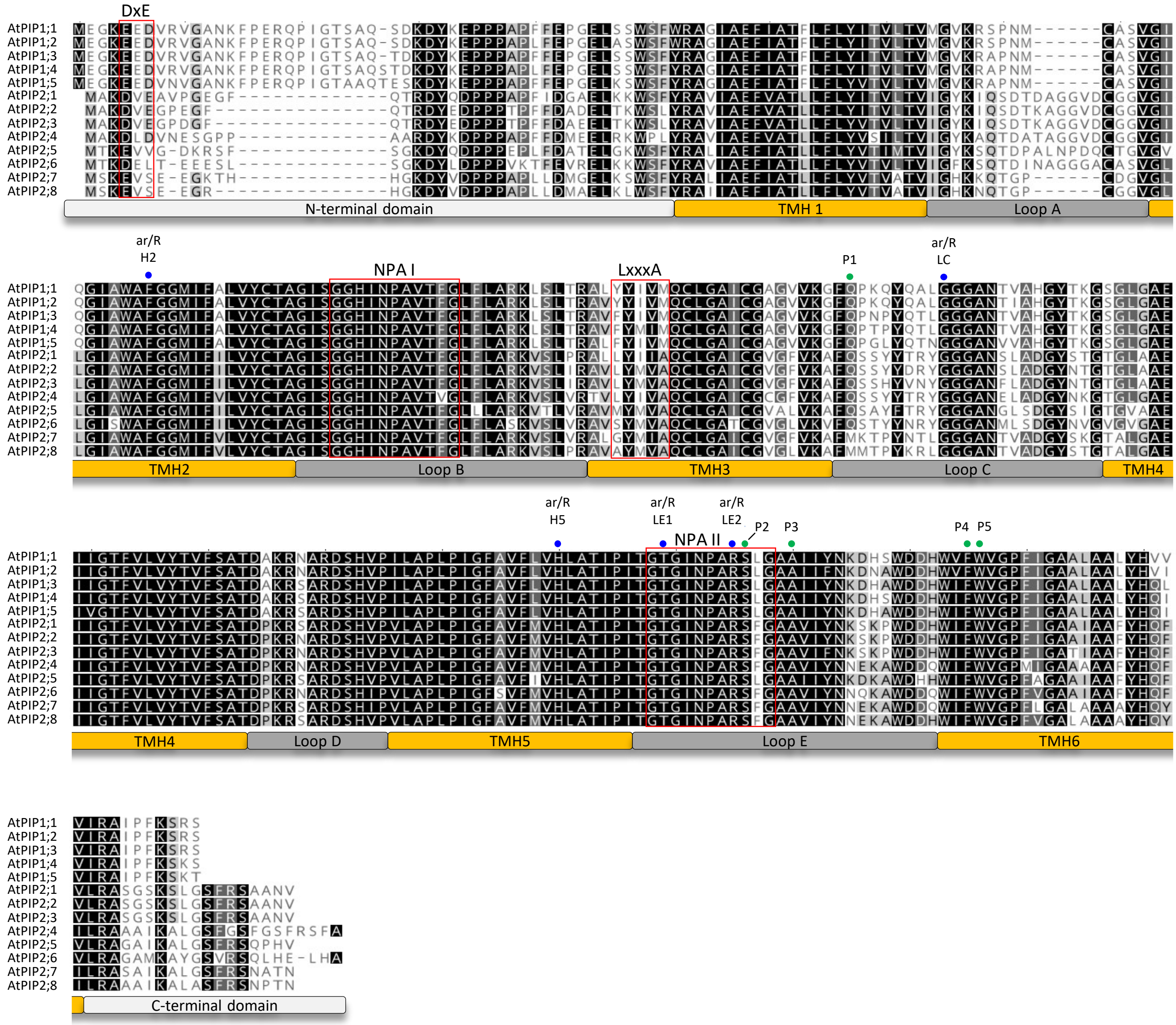

Supplemental Figure S10. AtPIP family protein sequence alignment. MUSCLE aligned sequences of all 13 AtPIP isoforms. The Transmembrane Helix domains (TMH1 to 6), Loop domains (Loop A-E) and N- and C-terminal domains are depicted below the sequences. Classic motifs that define substrate specificity (NPA motifs – red boxes; ar/R constriction point residues – blue dots; and Froger's positions P1-5 – green dots) and those that facilitate ER to PM trafficking (LxxA and DxE - red boxes) are labelled accordingly (also see Supplementary Table 2).

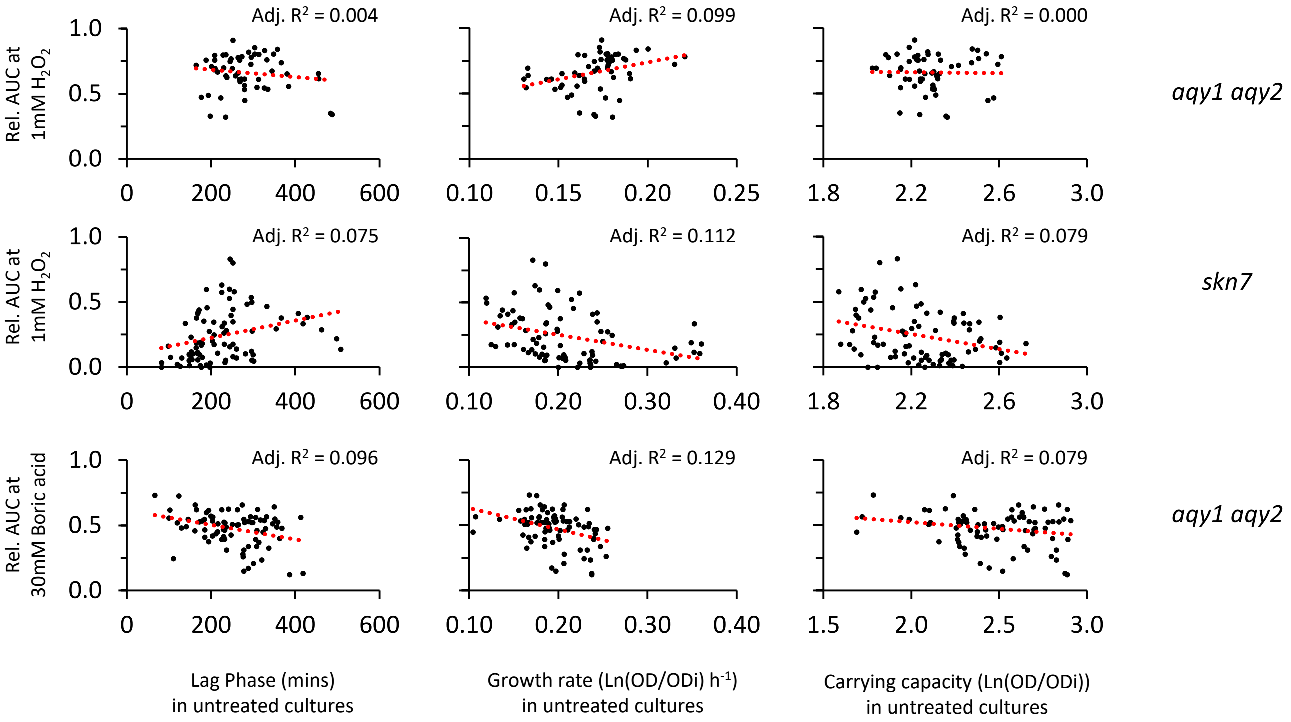

Supplemental Figure S11. Correlation analysis examining AtPIP induced changes in inherent yeast growth characteristics and possible indirect effects on response to treatments. No correlations were observed between treated ΔAUC values when compared against values for λ, μ, and κ from untreated cultures. This indicates that the differential growth responses of certain *AtPIP* yeast lines to specific treatments appears the direct result of AtPIP enhancement of membrane permeability to the tested substrate and not due to indirect effects through subtle changes to inherent growth characteristics.

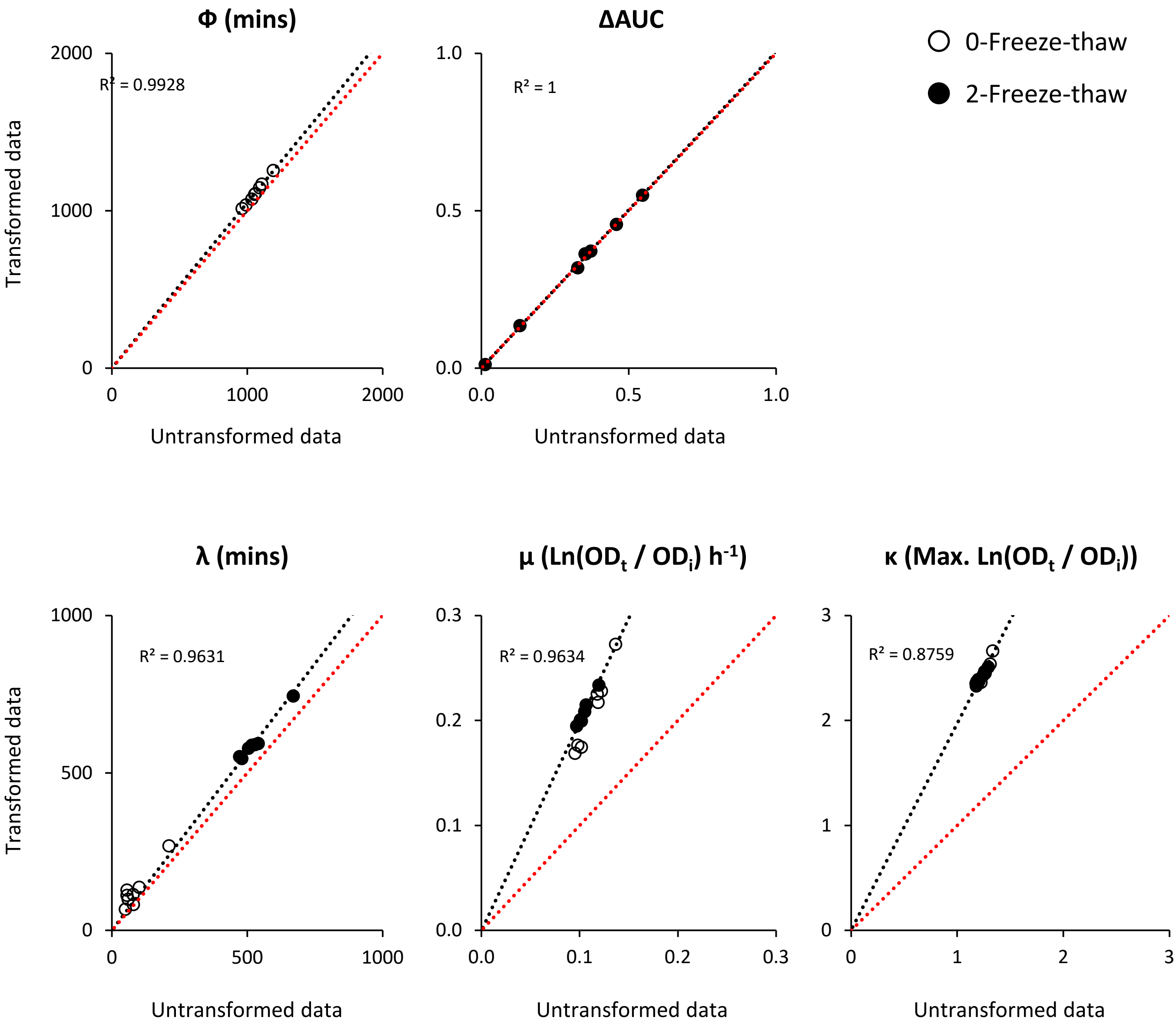

Supplemental Figure S12. Examination of growth curve transformation using PIP2-PIP1 co-expression for freeze-thaw treatment data presented in Figure 4D. Growth-curve transformation used to obtain corrected OD readings does not alter the measuring point ϕ or ΔAUC. As expected given the saturation limits of optical detection in spectrophotometers as a yeast population grows, severely underestimating 'true' ODs at higher cell densities. Key growth characteristics are thus incorrectly determined with λ being slightly and μ and κ greatly under-estimated when using raw 'recorded' (untransformed) OD reads.

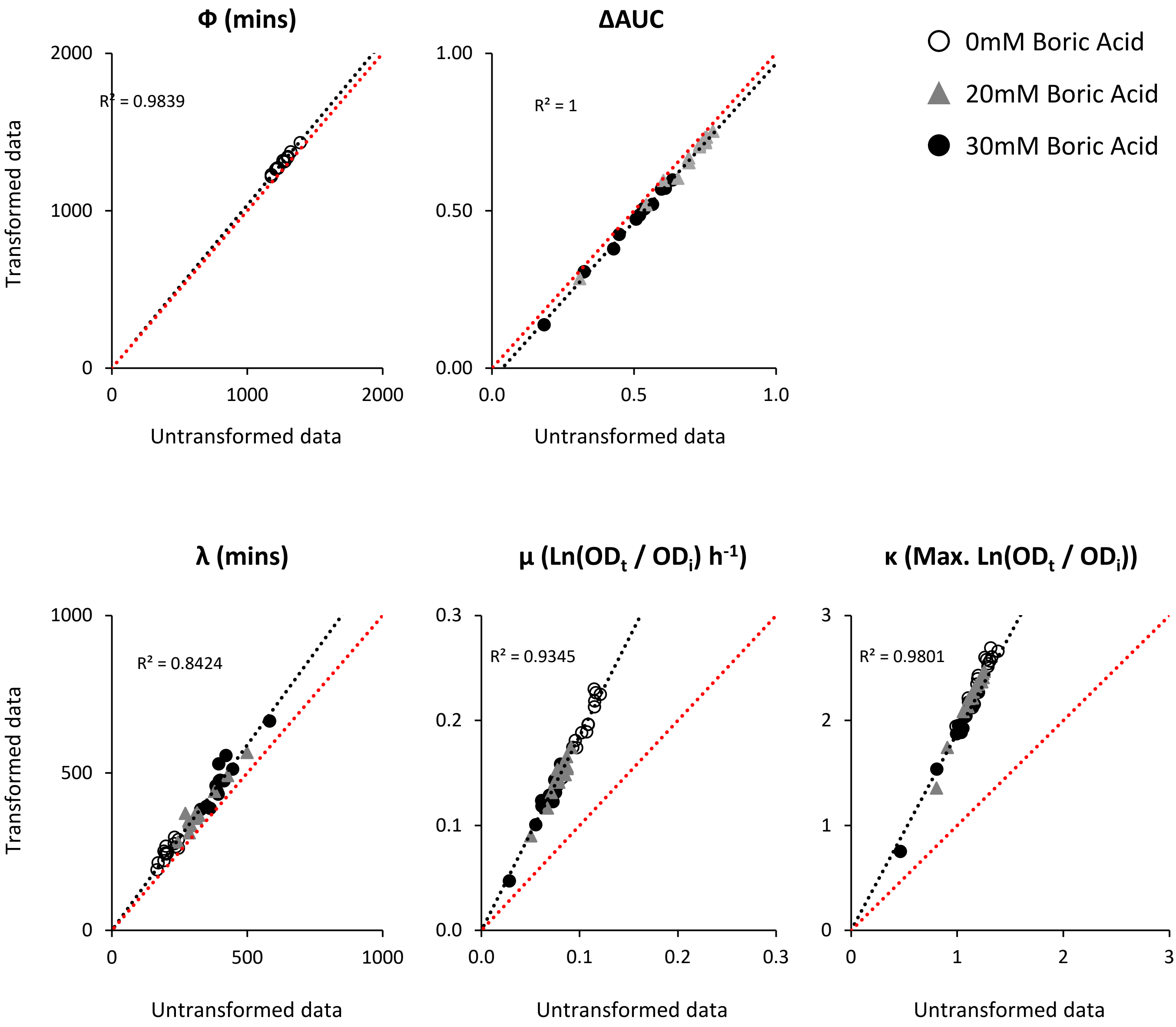

Supplemental Figure S13. Examination of growth curve transformation using AtPIP expressing yeast treated with boric acid presented in Figure 6 and Supplemental Figure S8. Growth-curve transformation used to obtain corrected OD readings does not alter the measuring point ϕ or ΔAUC. As expected given the saturation limits of optical detection in spectrophotometers as a yeast population grows, severely underestimating 'true' ODs at higher cell densities. Key growth characteristics are thus incorrectly determined with λ being slightly and μ and κ greatly under-estimated when using raw 'recorded' (untransformed) OD reads.

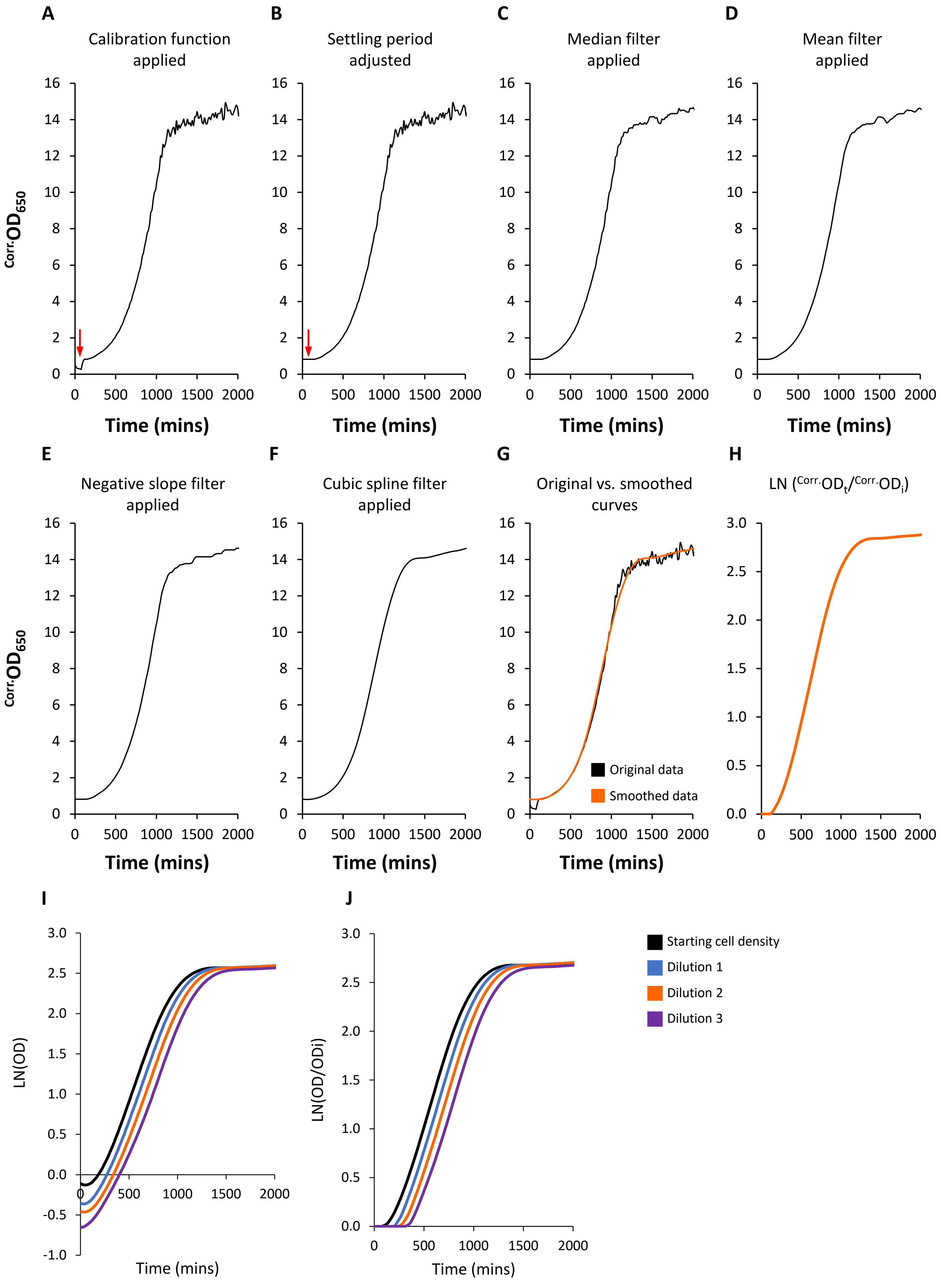

Supplemental Figure S14. Growth curve processing. A-F, Example showing the progressive application of filters to smooth the extracted growth curve data obtained from the micro-cultivation cultures. **G**, Overlay of the original corrected OD data and the smoothed data. **H**, The smoothed growth curve is log transformed (LN) and usually plotted as Ln($^{Corr.}OD_t$ / $^{Corr.}OD_i$). Growth curves obtained from 4 different dilutions: **I** LN ($^{Corr.}OD$), **J** LN($^{Corr.}OD_t$ / $^{Corr.}OD_i$).

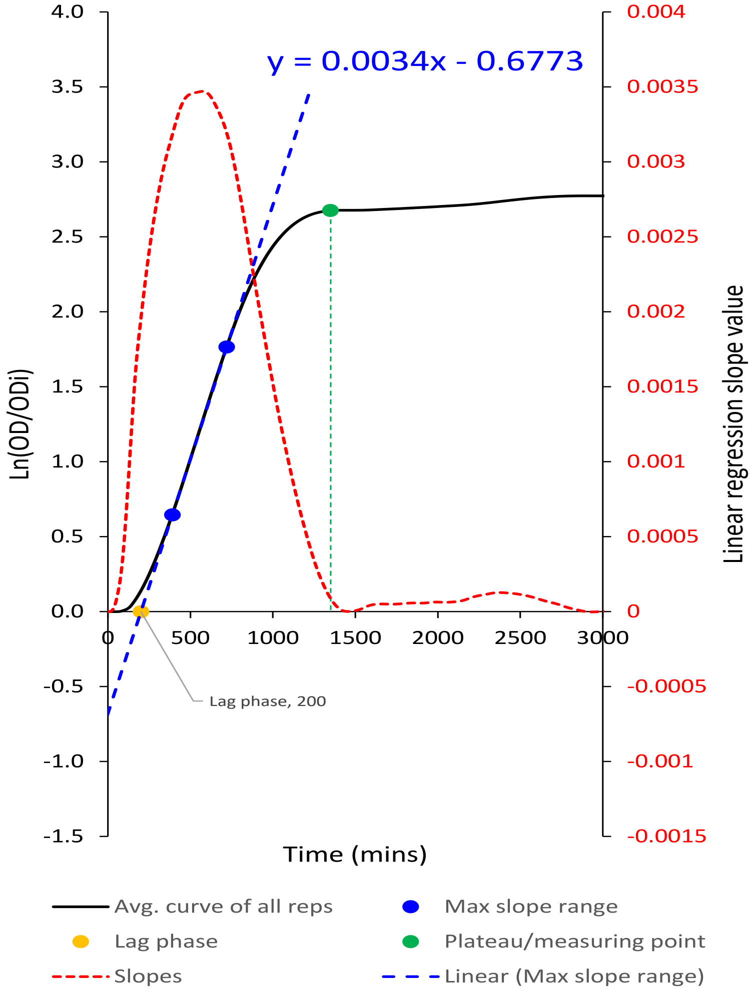

Supplemental Figure S15. From a processed growth curve, the following parameters were derived: μ, the maximum growth rate from the x coefficient of the blue linear equation centred on the peak of the red derivative curve, λ, the lag from the x intercept of the blue linear equation, and ϕ, the standardizing measuring point when the population growth rate drops below 5% of the maximum.
