# Supplementary material for: A high-throughput yeast approach to characterize aquaporin permeabilities: Profiling the Arabidopsis PIP aquaporin sub-family
Source: Front Plant Sci. 2023 Jan 19;14:1078220. doi: 10.3389/fpls.2023.1078220 (PMC9907170; doi:10.3389/fpls.2023.1078220)
Supplement: Supplementary file 3 [file Table_1.pdf]

Gene Name	TAIR Gene ID	Protein length (aa)	G+C nucleotide content (%)	Codon Adaptation Index value: *in Arabidopsis thaliana*	Codon Adaptation Index value: *in Saccharomyces cerevisiae*	Kozak related features: Nucleotide sequence surrounding mRNA AUG	Growth characteristics of AtPIP expressing yeast: Lag time (λ) (mins)	Growth characteristics of AtPIP expressing yeast: Max. growth rate (μ) $\ln(^{Corr.}OD_t / ^{Corr.}OD_i)\ h^{-1}$	Growth characteristics of AtPIP expressing yeast: Carrying capacity (κ) Max. $\ln(^{Corr.}OD_t / ^{Corr.}OD_i)$
AtPIP1;1	AT3G61430	286	50	0.80	0.72	AGA ACC **AUG** GAA	259 ± 24 ^{b,c,d}	0.189 ± 0.006 ^{b,c}	2.40 ± 0.05 ^{a,b,c}
AtPIP1;2	AT2G45960	286	50	0.80	0.71	AGA ACC **AUG** GAA	251 ± 14 ^{b,c,d}	0.184 ± 0.002 ^{b,c}	2.40 ± 0.06 ^{a,b,c}
AtPIP1;3	AT1G01620	286	52	0.76	0.69	AGA ACC **AUG** GAA	262 ± 15 ^{a,b,c,d}	0.195 ± 0.010 ^{a,b,c}	2.29 ± 0.05 ^{c,d}
AtPIP1;4	AT4G00430	287	51	0.78	0.70	AGA ACC **AUG** GAA	250 ± 16 ^{b,c,d}	0.177 ± 0.003 ^{c,d}	2.22 ± 0.03 ^{d}
AtPIP1;5	AT4G23400	287	49	0.79	0.72	AGA ACC **AUG** GAA	250 ± 19 ^{b,c,d}	0.178 ± 0.002 ^{c,d}	2.36 ± 0.05 ^{b,c,d}
AtPIP2;1	AT3G53420	287	50	0.76	0.71	AGA ACC **AUG** GCA	265 ± 12 ^{a,b,c,d}	0.183 ± 0.005 ^{b,c}	2.47 ± 0.06 ^{a,b}
AtPIP2;2	AT2G37170	285	51	0.73	0.68	AGA ACC **AUG** GCC	240 ± 13 ^{c,d}	0.199 ± 0.012 ^{a,b}	2.53 ± 0.08 ^{a}
AtPIP2;3	AT2G37180	285	51	0.73	0.67	AGA ACC **AUG** GCT	225 ± 14 ^{d}	0.181 ± 0.007 ^{b,c,d}	2.49 ± 0.07 ^{a,b}
AtPIP2;4	AT5G60660	291	51	0.73	0.69	AGA ACC **AUG** GCA	257 ± 24 ^{b,c,d}	0.164 ± 0.005 ^{d}	2.36 ± 0.07 ^{a,b,c,d}
AtPIP2;5	AT3G54820	286	52	0.76	0.70	AGA ACC **AUG** ACG	228 ± 31 ^{c,d}	0.140 ± 0.008 ^{e}	2.01 ± 0.07 ^{e}
AtPIP2;6	AT2G39010	289	51	0.74	0.68	AGA ACC **AUG** ACG	308 ± 34 ^{a,b}	0.187 ± 0.005 ^{b,c}	2.53 ± 0.07 ^{a}
AtPIP2;7	AT4G35100	280	50	0.81	0.74	AGA ACC **AUG** TCG	315 ± 24 ^{a}	0.212 ± 0.008 ^{a}	2.49 ± 0.05 ^{a,b}
AtPIP2;8	AT2G16850	278	49	0.78	0.73	AGA ACC **AUG** TCA	291 ± 25 ^{a,b,c}	0.201 ± 0.012 ^{a,b}	2.41 ± 0.07 ^{a,b,c}
empty vector	-	-		-	-	-	279 ± 20 ^{a,b,c,d}	0.195 ± 0.006 ^{a,b}	2.51 ± 0.05 ^{a,b}

Supplemental Table S1. *AtPIP* codon compatibility for heterologous expression in yeast and growth characteristics of *AtPIP* expressing yeast lines. The AtPIPs have similar good codon compatibility (CAI values and GC contents) for heterologous expression in yeast, with yeast CAI values similar to native yeast *AQP* genes (*AQY1*: 0.71 CAI and 51% G+C; *AQY2*: 0.79 CAI and 46% G+C). The upstream Kozak region (red nucleotides) were engineered as part of the vector construction. AtPIP expressing yeast cultured in standard selection medium (no treatment) show slight differences in growth characteristics between each other and empty vector control. Superscript letters denote statistical groupings, ANOVA with Tukey's test ($P < 0.05$). N ≥ 10 for each line.

Low Mid High
