# Supplementary material for: A high-throughput yeast approach to characterize aquaporin permeabilities: Profiling the Arabidopsis PIP aquaporin sub-family
Source: Front Plant Sci. 2023 Jan 19;14:1078220. doi: 10.3389/fpls.2023.1078220 (PMC9907170; doi:10.3389/fpls.2023.1078220)
Supplement: Supplementary file 4 [file Table_2.pdf]

			Length of protein domain (aa)													NPA I motif	NPA II motif	ar/R					Froger's positions					PM targeting		C-terminal domain
Protein Name	TAIR ID	Protein length (aa)	N-term	TM1	LA	TM2	LB	TM3	LC	TM4	LD	TM5	LE	TM6	C-term	LB	LE	H2	LC	H5	LE1	LE2	P1	P2	P3	P4	P5	DxE	LxxxA	
AtPIP1;1	AT3G61430	286	51	21	13	21	25	21	23	21	12	21	26	21	10	GGHINPAVTFG	GTGINPARSLG	F	G	H	T	R	Q	S	A	F	W	EED	YYIVM	IRAIPFKSRS
AtPIP1;2	AT2G45960	286	51	21	13	21	25	21	23	21	12	21	26	21	10	GGHINPAVTFG	GTGINPARSLG	F	G	H	T	R	Q	S	A	F	W	EED	YYIVM	IRAIPFKSRS
AtPIP1;3	AT1G01620	286	51	21	13	21	25	21	23	21	12	21	26	21	10	GGHINPAVTFG	GTGINPARSLG	F	G	H	T	R	Q	S	A	F	W	EED	FYIVM	IRAIPFKSRS
AtPIP1;4	AT4G00430	287	52	21	13	21	25	21	23	21	12	21	26	21	10	GGHINPAVTFG	GTGINPARSLG	F	G	H	T	R	Q	S	A	F	W	EED	FYMIM	IRAIPFKSKS
AtPIP1;5	AT4G23400	287	52	21	13	21	25	21	23	21	12	21	26	21	10	GGHINPAVTFG	GTGINPARSLG	F	G	H	T	R	Q	S	A	F	W	EED	FYIVM	IRAIPFKSKT
AtPIP2;1	AT3G53420	287	38	21	20	21	25	21	23	21	12	21	26	21	18	GGHINPAVTFG	GTGINPARSFG	F	G	H	T	R	Q	S	A	F	W	DVE	LYIIA	LRASGSKSLGSFRSAANV
AtPIP2;2	AT2G37170	285	36	21	20	21	25	21	23	21	12	21	26	21	18	GGHINPAVTFG	GTGINPARSFG	F	G	H	T	R	Q	S	A	F	W	DVE	LYMVA	LRASGSKSLGSFRSAANV
AtPIP2;3	AT2G37180	285	36	21	20	21	25	21	23	21	12	21	26	21	18	GGHINPAVTFG	GTGINPARSFG	F	G	H	T	R	Q	S	A	F	W	DVE	LYMVA	LRASGSKSLGSFRSAANV
AtPIP2;4	AT5G60660	291	38	21	20	21	25	21	23	21	12	21	26	21	22	GGHINPAVTVG	GTGINPARSFG	F	G	H	T	R	Q	S	A	F	W	DLD	LYIVA	RAAAIKALGSFGSFGSFRSFA
AtPIP2;5	AT3G54820	286	37	21	20	21	25	21	23	21	12	21	26	21	18	GGHINPAVTFG	GTGINPARSLG	F	G	H	T	R	Q	S	A	F	W	EEV	MYMVA	RAGAIKALGSFRSQPHV
AtPIP2;6	AT2G39010	289	37	21	20	21	25	21	23	21	12	21	26	21	21	GGHINPAVTFG	GTGINPARSFG	F	G	H	T	R	Q	S	A	F	W	DEL	SYMVA	RAGAMKAYGSVRSQLHELHA
AtPIP2;7	AT4G35100	280	37	21	14	21	25	21	23	21	12	21	26	21	18	GGHINPAVTFG	GTGINPARSFG	F	G	H	T	R	M	S	A	F	W	EVS	GYMIA	RASAIKALGSFRSNATN
AtPIP2;8	AT2G16850	278	35	21	14	21	25	21	23	21	12	21	26	21	18	GGHINPAVTFG	GTGINPARSFG	F	G	H	T	R	M	S	A	F	W	EVS	AYMVA	RAAAIKALASFRSNPTN

Supplemental Table S2. Protein domain lengths and amino acid composition of AtPIPs at known substrate selectivity positions and other important motifs. The N-terminal domain of AtPIP1s is distinctly longer than those of AtPIP2s, whereas the C-terminal domain is longer in AtPIP2s than AtPIP1s. More serine residues are present in the C-terminal domain of AtPIP2s than AtPIP1s (bold black), with two positions in the AtPIP2s likely to be phosphorylation targets (bold blue). The extracellular loop A domain is longer in AtPIP2;1 to 2;6 isoforms. Classic motifs that define substrate specificity (i.e. NPA motifs, ar/R constriction point, and Froger's positions) are nearly identical, with only seemingly minor conserved differences. There is substantial variation in the motifs associated with PM targeting between the AtPIP isoforms.
